# Dramatic loss of microbial viability in bentonite exposed to heat and gamma radiation: implications for deep geological repository

**DOI:** 10.1007/s11274-024-04069-w

**Published:** 2024-07-11

**Authors:** Deepa Bartak, Šárka Šachlová, Vlastislav Kašpar, Jakub Říha, David Dobrev, Petr Večerník, Veronika Hlaváčková, Michaela Matulová, Kateřina Černá

**Affiliations:** 1 Institute for Nanomaterials, Advanced Technologies and Innovation, Technical University of Liberec, Bendlova 7, Liberec, 460 01 Czech Republic; 2 Disposal Processes and Safety, ÚJV Řež, a. s., Hlavní 130, Husinec 250 68 Czech Republic; 3 Radioactive Waste Repository Authority, SÚRAO, Dlážděná 6, Prague 11000 Czech Republic

**Keywords:** Bentonite buffer, Elevated temperature, Gamma radiation, Microbial limiting factors, Extremophiles, Radioactive waste disposal, Deep geological repository

## Abstract

Bentonite is an integral part of the engineered barrier system (EBS) in deep geological repositories (DGR) for nuclear waste, but its indigenous microorganisms may jeopardize long-term EBS integrity. To predict microbial activity in DGRs, it is essential to understand microbial reactions to the early hot phase of DGR evolution. Two bentonites (BCV and MX-80) with varied bentonite/water ratios and saturation levels (compacted to 1600 kg.m^− 3^ dry density/powder/suspension), were subjected to heat (90–150 °C) and irradiation (0.4 Gy.h^− 1^) in the long-term experiments (up to 18 months). Molecular-genetic, microscopic, and cultivation-based techniques assessed microbial survivability. Exposure to 90 °C and 150 °C notably diminished microbial viability, irrespective of bentonite form, with negligible impacts from irradiation or sample type compared to temperature. Bentonite powder samples exhibited microbial recovery after 90 °C heating for up to 6 months but not 12 months in most cases; exposure to 150 °C had an even stronger effect. Further long-term experiments at additional temperatures combined with the mathematical prediction of temperature evolution in DGR are recommended to validate the possible evolution and spatial distribution of microbially depleted zones in bentonite buffer around the waste canisters and refine predictions of microbial effects over time in the DGR.

**Supplementary Information:**

The online version contains supplementary material available at 10.1007/s11274-024-04069-w.

## Introduction

The use of deep geological repositories (DGRs) is a widely accepted method for disposing of high-level radioactive waste in many countries (OECD and Nuclear Energy Agency 2006; IAEA 2009), including the Czech Republic (Havlová et al. 2020; Kumpulainen et al. 2022). This approach relies on a multi-barrier system, incorporating a natural barrier (host rock) and an engineered barrier system (EBS), i.e., a metal canister containing the nuclear waste enclosed in bentonite buffer, to safeguard the repository’s integrity (OECD and Nuclear Energy Agency, 2008, 2006, 2003). The goal is to establish optimal physicochemical conditions, ensuring the repository securely contains the waste, retains and retards radionuclides, and controls generated gas transport (Posiva Oy 2017). Highly compacted bentonite clay is a crucial element of the EBS system, serving as a buffer around waste canisters and as backfill material in disposal tunnels (Posiva Oy 2017; Birgersson et al. 2017). Its properties, including its self-sealing ability, low hydraulic conductivity, high sorption capacity, swelling capacity, thermal conductivity, and long-term stability, contribute to preventing the passage of corrosive groundwater components, offering mechanical protection, retarding radionuclide migration, and averting mobilization of corrosion products (SKB 2010a; Posiva Oy 2017).

Being a natural material, however, bentonite contains a diverse spectrum of indigenous microorganisms (Vachon et al. 2021), including sulfate-reducing bacteria (SRB), nitrate-reducing bacteria (NRB), iron-reducing bacteria (IRB), acetogens, methanogens, and various other microorganisms (Stroes-Gascoyne 2010; Urios et al. 2013; Bengtsson and Pedersen 2017; Liu et al. 2019; Burzan et al. 2022; Mijnendonckx et al. 2022). The natural occurrence of spore-forming bacteria in bentonite is of particular concern (Haynes et al. 2018) as they are capable of surviving extreme conditions (Nicholson et al. 2000; Haynes et al. 2018), and remaining dormant until conditions become more favorable (Nicholson et al. 2000; Stroes-Gascoyne 2010). Uncontrolled microbial activity within the DGR could lead to adverse effects such as microbially-influenced corrosion (MIC), bentonite illitization (smectite alteration), gas production causing pressure built-up, and/or potential radionuclide migration, causing safety and integrity concerns (Kim et al. 2004; Mulligan et al. 2009; Beaton et al. 2019; Lopez-Fernandez et al. 2021a, b; Hall et al. 2021; Shrestha et al. 2021). Survival of SRB is of particular significance in MIC as hydrogen sulfide production can corrode metal canisters, where copper-based canisters are especially threatened by this process (El Mendili et al. 2013; Enning and Garrelfs 2014; Bengtsson and Pedersen 2017).

Following high-level nuclear waste deposition, a repository undergoes four phases of evolution, transitioning from a warm, aerobic phase through a long-term cooling period under anaerobic conditions. The initial stage involves aerobic conditions with a high gamma radiation dose rate lasting several months. Subsequently, a hot and dry phase lasts a decade, depending on the bentonite’s initial moisture content, dry density, and host rock type (King 2009; King et al. 2017). In the Switzerland concept, it is expected that the temperature on the canister surface can reach up to 150 °C and the temperature inside the buffer material can thus exceed 100 °C in the initial hot phase due to the very low thermal conductivity of dried bentonite (Johnson et al. 2002). On the other hand, in the KBS-3 and KBS-3 based Czech repository design, the canister surface temperatures and, thus, bentonite maximum temperatures should not exceed 100 °C/95°C respectively (SKB 2010b; Hausmannová et al. 2023). To achieve this, bentonite buffer thus needs to have sufficient thermal conductivity (function of dry density and the moisture content) to avoid the overheating of the canister with the spent fuel and also to ensure that retardation and geotechnical properties of the bentonite are not lost due to the increased temperature (Svoboda et al. 2022). During the third stage, the DGR transitions from a dry state to a fully-saturated state, while the final, fourth stage is characterized by cool and anoxic conditions (King 2009; King et al. 2017).

During the early repository phase, the prevailing physicochemical conditions (elevated temperatures, peak irradiation levels, and low water saturation) are assumed to create an environment unfavorable for microbial activity (Motamedi et al. 1996; Stroes-Gascoyne and West 1997; Aoki et al. 2010; Gregory et al. 2024). High temperatures, for example, can exacerbate the impact of radiation on microorganisms as heat accelerates chemical reactions and increases radiation damage (Stroes-Gascoyne et al. 1995; Todoriki et al. 2000). The detrimental effects of high temperatures on microbial survivability will primarily be due to protein denaturation and disruption of cell membranes (Morozkina et al. 2010); however, certain microorganisms, such as SRB, have shown tolerance to high temperatures (Masurat et al. 2010a; Martinez-Moreno et al. 2024) and can enter dormancy to endure extreme conditions (Gilmour et al. 2022; Butterworth et al. 2023). Spore formers, such as *Bacillus subtilis* and *Desulfotomaculum nigrificans*, have been shown to survive for 15 months at temperatures ranging from 50 to 70 °C in bentonite buffer under repository-relevant conditions (Pedersen 2000). The overall temperature threshold for survivability of common microbes (aside from extremophile prokaryotes) is believed to be 121 °C (Kashefi and Lovley 2003). Yet, microbial analyses of bentonite from one of our long-term experiments indicated that microorganisms can survive in powdered Czech calcium-magnesium bentonite (BCV), even when heated to 150 °C for 6 or even 12 months (Kašpar et al. 2021). However, these data still need to be verified through independent studies as the experiment was not initially designed for microbiological application, and contamination is a possibility. Similarly, ionizing radiation emits high energy, potentially threatening microorganisms (Kaminski et al. 2021). Radiation causes oxidative damage to bacterial DNA, lipids, proteins, and other metabolites (Du and Gebicki 2004; Ghosal et al. 2005; Daly et al. 2007; Reisz et al. 2014), leading to biological or chemical changes within the cell and its components, ultimately resulting in cell death (Krisko and Radman 2010; Bhana et al. 2013). While most microorganisms are sensitive to elevated radiation levels, certain extremophiles, such as *Deinococcus radiodurans* (Mattimore and Battista 1996), and spore-formers exhibit remarkable resistance to radiation (Bruhn et al. 1999). Notably, some microorganisms frequently present in bentonite, such as *Bacillus* and *Acinetobacter*, also exhibit high radiation resistance (Stroes-Gascoyne et al. 1995; Todoriki et al. 2000). The simultaneous application of heat and radiation can stimulate biphasic survival responses, indicating synergistic effects between heat and radiation (Stroes-Gascoyne and West 1997).

Numerous studies have described microbial activity under conditions simulating the later colder and saturated phase of DGR evolution using either dispersed (e.g. Matschiavelli et al. 2019; Povedano-Priego et al. 2023; Park et al. 2024) or compacted bentonite samples (e.g. (Pedersen 2010; Bengtsson and Pedersen 2017; Bengtsson et al. 2017; Engel et al. 2019; Vachon et al. 2021). However, for reliable predictions of long-term microbial effects in DGRs, it is crucial to also assess changes in microbial survivability during the initial hot phase. This can be only achieved by evaluation of microbiological activity in heated bentonite experiments as performed, e.g., by (Aoki et al. 2010; Engel et al. 2019; Martinez-Moreno et al. 2024). Similar studies are, however, still rather scarce. As part of the European Joint Programme on Radioactive Waste Management (EURAD), work package ‘Concord (Container Corrosion under Disposal conditions)’, we focused on microbial response to the combined effects of heat (90–150 °C) and gamma radiation (≈ 0.4 Gy.h^− 1^ dose rate) on bentonite (BCV and MX-80) under anaerobic conditions in the 18-months long experiment, emphasizing microbial potential to regenerate from dormant stages post-treatment. Our study approach included both cultivation-dependent and cultivation-independent methods and included different saturation levels and bentonite/water/air ratios (powder/suspension/compacted bentonite) to check for their possible effect on microbial survivability.

## Materials and methods

### Experimental setup

Three sets of experiments (labeled A, B, and C) were conducted to analyze the effect of long-term exposure to heat and irradiation on microbial survivability in bentonites (hereon referred to as the long-term experiment). Experimental set A was designed to mimic a fully saturated compacted bentonite system during the later DGR hot phase, while experimental set B simulated a non-saturated system post-closure. The experimental set C then completed sets A and B by targeting two possible extremes in the water-saturation state of bentonite - bentonite suspension and dry powder. Additionally, a second experiment was conducted to obtain more detailed information on the effect of length of heat exposure on microbial survivability (hereon referred to as the additional experiment).

Both experiments used Czech calcium-magnesium bentonite (BCV) provided by KERAMOST, Plc. (Czech Republic) and reference sodium bentonite (MX-80) provided by the Research Centre for Energy, Environment and Technology (CIEMAT, Spain).

#### Long-term experiment

**Fig. 1:**
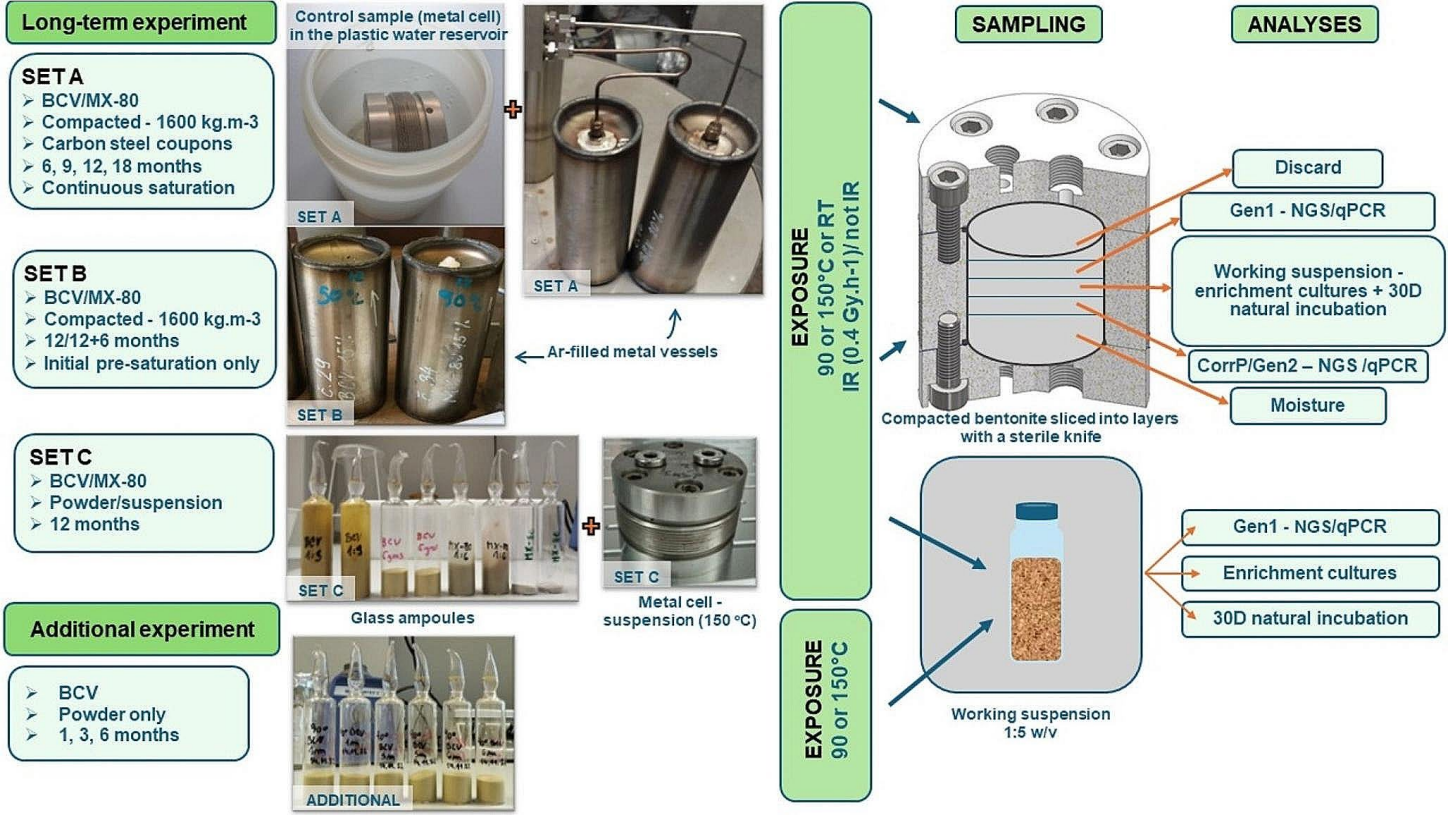
Illustration of the experimental set-up showing the long-term experiment depicting sets **A** (with carbon steel coupons), **B**, and **C** using BCV/MX-80 bentonite in compacted, powdered, suspension form exposed to heat treatments at 90 °C and 150 °C, with or without irradiation, for exposure durations of 6, 9, 12, and 18 months along with an additional experiment where BCV powder underwent heat treatments at 90–150 °C for 1, 3, and 6 months

Experimental set A consisted of fully saturated BCV and MX-80 compacted bentonite. Samples were compacted in metal cells (Fig. 1) to a dry density of 1600 kg.m^− 3^ from bentonite partially pre-saturated with sterile distilled water (DW) before compaction (15% relative water content for samples heated to 150 °C and 20% for samples heated to 90 °C). The presaturation level was set to align with the past experiments (20% for samples heated at 90 °C) and other project partners (15% saturation, the rest of the samples including set B). Unlike sets B and C, the compacted bentonite samples of set A also contained carbon steel coupons prepared from a reference material for the Czech canister concept (S355J2H; Pospiskova et al. 2017), supplied by Škoda Auto a.s. (Czech Republic). The corrosion data will be published in a separate article to estimate the corrosion rate under the studied conditions. The prepared partially saturated samples were then connected to the saturation system filled with synthetic granitic water (SGW3; Červinka et al. 2018), which continuously saturated the samples under the pressure of 5 MPa and sealed them in air-tight argon-filled steel vessels to maintain an anaerobic atmosphere during the experiment (Fig. 1). The samples were exposed to heat (90–150 °C) or a combination of heat (90–150 °C) and irradiation (^60^Co source) at an approx. dose rate of 0.4 Gy.h^− 1^. Laboratory controls not exposed to either heating or irradiation were included to facilitate comparison between treated and non-treated samples. Control bentonite samples emerged in plastic saturation reservoirs containing sterile DW and were continuously saturated in the anaerobic box at laboratory temperature (Fig. 1).

For BCV, the exposure times (to all the conditions) were 6-, -9-, 12-, and 18 months at 150 °C and 9-, 12-, and 18 months at 90 °C. For MX-80, exposure was limited to 18 months only.

Samples in experimental set B comprised pre-saturated (15% moisture content in all the samples) compacted bentonite (BCV/MX-80, 1600 kg.m^− 3^ dry density). However, contrary to set A, these samples did not contain metal coupons and were not continuously saturated during the experiment. The samples were sealed in air-tight argon-filled steel vessels to maintain an anaerobic atmosphere and exposed to either heat (90–150 °C) or a combination of heat and irradiation (0.4 Gy.h^− 1^) for 12 months (MX-80 only temperature 90 °C). Control samples were prepared similarly to the treated samples but kept at room temperature. After 12 months of exposure, half of the BCV samples (both treated and controls) were dismantled and analyzed directly, and the second half was placed in plastic saturation reservoirs filled with DW, similar to control samples in Set A (Fig. 1) and incubated in a saturated state for a further 6 months in an anaerobic chamber at room temperature to detect possible microbial recovery in treated samples.

Experimental set C comprised bentonite (BCV/MX-80) as a dry powder in sterile DW at 1:3 (BCV)/1:6 (MX-80) weight: volume (w/v) ratio, representing two possible extremes in terms of water availability in a bentonite environment. The w/v ratios differed in both bentonites because of the different swelling capacities of these materials, and the chosen ratio resulted in roughly similar suspension consistency necessary for subsequent sample handling. All the bentonite powder samples, suspensions heated at 90 °C, and suspension controls were prepared in sealed glass ampoules to maintain anoxic conditions during the incubation (Fig. 1). The suspension samples exposed to 150 °C were prepared in pressure-resistant metal cells for compacted bentonite enclosed in in Ar-filled metal vessel similarly to sets A and B. Prepared samples were exposed to heat (90–150 °C) or a combination of heat and irradiation (0.4 Gy.h^− 1^) under anoxic conditions for 12 months, except for suspensions heated at 150 °C (both irradiated and non-irradiated), where only 1-month heating was possible due to technical issues. Supplementary Table S1 summarizes the list of samples and an overview of all the conditions used in each set.

#### Additional experiment

Based on the first sampling data after 6 m from the set A of the long-term experiment, a simple additional experiment was performed to distinguish better the time effect of heat exposure on bentonite in its natural dry state. This experiment comprised BCV powder solely heated to either 90–150 °C for shorter periods of 1, 3, and 6 months (Fig. 1). Bentonite in the dry powder form was chosen because the highest chance of survivability was expected here based on our previous experimental data (Kašpar et al. 2021). All samples were sealed in glass ampoules to maintain anoxic conditions and exposed to the desired temperature in a hot oven.

### Sample processing

The compacted bentonite samples from set A were disassembled in a glove box under an Ar atmosphere (O_2_ concentration < 0.1 ppm). In this case, maintaining fully sterile conditions during sampling was impossible as the priority was retaining the samples under anaerobic conditions throughout the experiment for subsequent corrosion analyses (data to be published separately). Samples from sets B and C and from the additional experiment were sampled in a sterile laminar box as, in this case, sample sterility was a priority.

In sets A and B, the compacted bentonite samples were expressed from the metal cell by a piston and sliced on a sterile petri dish using a sterile knife. Slices that were in contact with the metal cell lids with saturation ports were discarded, and from the remaining inner part of the bentonite block, samples were taken for genetic analysis (Gen1), cultivation analysis, and moisture content estimation. From set A, samples were also taken from the corrosion layers (CorrP) formed close to the metal coupons for genetic analysis. In some cases, corrosion product sampling was not possible; in that case, another bentonite sample was taken from the more distal part of the cell (Gen2). In the case of set C and the additional experiment, the glass ampoules were carefully broken open in a laminar flow box after the outer surface of the glass had been sterilized, and the samples were carefully transferred to sterile plastic tubes for further analysis.

For the subsequent analyses, all samples (compacted bentonite, powder, and suspension) were first suspended in sterile deionized water to obtain a working suspension with an approx. 1:5 ratio w/v (bentonite to water) for BCV and 1:8 for MX-80. These ratios were chosen based on suspension viscosity and subsequent ease of handling. From each working suspension, (1) six enrichment culture samples in total were prepared by transferring 0.5 ml of working suspension to 14 ml of media. R2A broth (M1687; Himedia, India) was used for culturing aerobic (R2A_AE) and anaerobic heterotrophic microorganisms (R2A_ANA) in duplicate for each. Postgate medium (PGM) (M803; Himedia, India) for culturing anaerobic SRB, again in duplicate. These cultivations were then incubated for 1 week (R2A - aerobic), 3 weeks (R2A - anaerobic), and 5 weeks (PGM) at 30 °C, respectively. (2) An additional 10 ml of the working suspension was taken and subjected to 30-day (30D) natural incubation in an anaerobic chamber containing a 6% H_2_ + 94% Ar atmosphere at room temperature. This natural incubation was undertaken to simulate more realistic culture conditions and to overcome the primary limitation of culture-based methods, i.e. high selectivity. At the end of the incubation time, all media cultures and 30D natural incubations were first checked microscopically for the presence of living cells, after which all samples were centrifuged at 11,000 x g for 10 min, and the resulting pellets were stored in a freezer (-20 °C) for subsequent DNA extraction and genetic analysis. In the case of the compacted bentonite controls from set A and samples from set B (re)saturated in plastic reservoirs, the water from these reservoirs (Water) was filtered through 0.22 μm Sterivex filters and used for subsequent DNA extraction and genetic analysis. The aim of this analysis was to assess the diversity of bacteria in the saturation solution and its similarity to microbial diversity in compacted bentonite.

### Genetic analysis

#### DNA extraction and qubit measurement

Two different extraction methods were used on the samples. Fresh bentonite samples (Gen1/Gen2/CorrP) and 30D natural incubations were extracted (approx. 5 g wet weight) using the DNeasy PowerMax Soil Kit (Qiagen, Germany), following the manufacturer’s protocol but finishing with 1 mL of DNA extract. This 1mL extract was subsequently purified and concentrated to a final volume of 50 µL using the Genomic DNA Clean & Concentrator kit (Zymo Research, USA), following the manufacturer’s protocol. Pellets from the culture samples and DNA from the Sterivex filters were extracted using the DNeasy PowerWater kit (Qiagen, Germany), following the manufacturer’s protocol. As microbial DNA is generally highly susceptible to environmental contamination during sample processing, a kit (negative) control sample without the input matrix was processed in the same way as the actual samples alongside each extraction batch to uncover contamination arising during DNA isolation, either from the environment (laboratory background) or from the kits themselves (kit contamination).

#### Relative quantification by qPCR

Quantitative PCR (qPCR) on a LightCycler^®^ 480 system (Roche, Switzerland) was used to monitor changes in the relative abundance of total bacterial biomass, using the universal primers U16SRT-F (5′-ACTCCTACGGGAGGCAGCAGT-3′) and U16SRT-R (5′-ATTACCGCGGCTGCTGGC-3′) to target all bacteria encoding the V3 region of the 16S rRNA gene (Clifford et al. 2012). Preparation of the qPCR reaction mix and PCR cycler conditions were as described by (Shrestha et al. 2022), with two technical replicates analyzed for each sample. The sample was reanalyzed if the resulting differences between replicate quantification cycle values (Cq) were not consistently < 0.5. Non-template control and positive control samples were also included in each qPCR run to check the background detection limit and signal reproducibility between runs.

Potential ongoing microbial proliferation in bentonite and culture samples was detected through a decrease in normalized by sample mass Cq values (= increase in 16S rRNA gene copy numbers), Supplementary Table S2. The Cq positivity threshold had to be estimated for each matrix separately. For this, all samples with negative microscopic results for all enrichment cultures (see below) and with Cq values close to the no template control (NTC) (Cq_NTC_ = 30) were selected and used to calculate mean Cq values (Cq_Avg_) together with their standard deviations (SD_Avg_) independently for each bentonite and matrix type (BCV/MX-80 bentonite, R2A, and PGM medium). The conservative threshold of positivity was set as Cq_Avg_ − 3 × SD_Avg_ for each bentonite type and matrix. All Cq values lower than this threshold were considered positive (Supplementary Tables S3 and S4).

#### Library preparation and 16S rRNA gene sequencing

All bentonite samples, together with all positive culture samples, were sequenced. For samples with a low DNA yield (< 0.5 ng/µL and Cq values > 18), two PCR reactions were performed with standard and barcoded fusion primers, while only one PCR reaction containing barcode fusion primers was performed in the case of samples with higher DNA concentrations (≥ 0.5 ng/µL and Cq values < 18). PCR conditions were as follows: an initial cycle at 95 °C for 3 min, followed by 10 (first PCR)/35 (second PCR) cycles at 98 °C for 20 s, 50 °C for 15 s, and 72 °C for 45 s, with a final extension at 72 °C for 1 min. The thermocycling conditions were the same for the first and second PCR reactions, except for the number of cycles. For both PCR runs, EliZyme HS HIFI MIX polymerase (Elizabeth Pharmacon, Czech Republic) and the universal primers 515F (Dowd et al. 2008) and 802R (Claesson et al. 2010) were used for amplification of the hypervariable V4 region of the 16S rDNA gene. The size of the amplicon was kept below 400 bp to cover as much microbial diversity as possible (Němeček et al. 2017). The amplified PCR product was then purified using the Agencourt Ampure XP system at a 50:50 ratio of PCR product: Ampure XP paramagnetic beads (Beckman Coulter, USA), following the manufacturer’s protocol. The concentration of purified PCR product was measured using a Qubit 2.0 fluorimeter (Life Technologies, USA). Finally, barcode-tagged amplicons from different samples were mixed at equimolar concentrations (25nM solution in 20 µL), and next-generation sequencing (NGS) was performed on an Ion Torrent Genexus system (Thermo Fisher Scientific, USA) using the Genexus sequencing kit combo with the Ion GX5 chip (Thermo Fisher Scientific, USA), following the manufacturer’s instructions.

#### Bioinformatics and statistical analysis

The data obtained were analyzed using the QIIME 2 2021.8 software package (Bolyen et al. 2019). First, the raw sequence data were demultiplexed and quality filtered using the q2-demux plugin, then denoising was performed with DADA2 (via q2-dada2; Callahan et al. 2016). Taxonomy was assigned to amplicon sequence variants (ASVs) using the q2-feature-classifier (Bokulich et al. 2018) and classified through classify-sklearn naive Bayes against the Silva 138 database (Quast et al. 2013). An artificial MOCK community sample was used to evaluate the accuracy of classification and the QIIME 2 outputs processed using the phyloseq R package (McMurdie and Holmes 2013). Mitochondria and chloroplasts were then removed from the dataset. In the bentonite and culture samples from the long-term experiment, statistical identification and removal of contaminant sequences was undertaken using the Decontam package v1.20.0 (Davis et al. 2018), based on DNA concentration and sequenced negative control samples (combined method option). The list of contaminants detected, together with the original data, are presented in supplementary Tables S5 (bentonite control), S6 (BCV cultivations), and S7 (MX-80 cultivations). Decontamination was not applicable in the additional experiment samples as too few positive samples were identified; consequently, the samples are presented in their original composition.

ANOVA and Principal Coordinates Analysis (PCoA) were used to compare bacterial communities across different bentonite samples, with the Bray-Curtis distance metric employed to measure differences between communities based on their relative abundances without rarefaction. ANOVA was used to test for the effect of treatment (treated/control samples), experimental set (A/B/C), sample type (fresh bentonite/30D/CorrP), and irradiation (irradiated/non-irradiated) on differences in microbial composition. Additionally, taxonomy bubble plots were created using the same relative abundances but only including bacteria with a mean relative abundance > 0.01.

### Cell extraction and microscopy analysis

Cell extraction followed by LIVE/DEAD (L/D) staining was performed on 30D natural incubations using 1 mL of suspension, as described in (Hlavackova et al. 2023). L/D staining was also applied to detect the presence of living and dead cells in culture samples, using 8 µL from each culture sample mixed with 4 µL of L/D BacLight ™ Bacterial Viability Kit fluorescent dye (Thermo Fischer Scientific, USA). The stained sample was incubated in the dark for 15 min before observing under a Zeiss Axio Imager M2 epifluorescence microscope (Carl Zeiss, Germany), using the AxioVision (AxioVs40 × 69 V v.4.9.1.0) imaging software program (Carl Zeiss, Germany).

## Results

### Long-term experiment

#### Cultivations

The enrichment culture growth was evaluated using microscopical (L/D) and genetic analyses (qPCR). In general, the qPCR results aligned well with the microscopy observations. In the case of disparities, the qPCR results were considered more relevant, as microscopy can easily indicate false negative results in bentonite environments.

Cultivations obtained from the heat/IR-treated BCV and MX-80 samples during long-term experiments A, B, and C only displayed microbial proliferation in a few culture samples across all three cultivation conditions (Table 1). Most of these positive culture samples from the treated samples belonged to the experimental set A, which was dismantled in semi-sterile conditions. Positive culture samples were detected in samples exposed to 150 °C and 90 °C and in irradiated and non-irradiated samples.

**Table 1:** Summary result table: qPCR (cq values) and microscopy (L/D) results obtained from the long-term experiment (sets A, B, and C). Sample names indicate the treatment and bentonite type (BCV/MX-80 bentonite): cBCV/MX-80 = compacted samples, pBCV/MX-80 = powder samples, sBCV/MX-80 suspension samples, C = sample number, 6–18 m = exposure duration, T90/150 = temperature applied, IR = irradiated samples, control = control samples. MC = metal coupons, CS = continuous saturation, DD = dry density, w_start_ = initial moisture content, w_final_ = final moisture content, +/- presence or absence of microbial growth, n/a = not analyzed. Gen (1/2) = fresh bentonite samples for genetics, 30D = 30-day naturally incubated samples in suspended form, CorrP = corrosion product, water = reservoir water, R2A_AE (1/2) = duplicate R2A aerobic enrichment culture, R2A_ANA (1/2) = duplicate R2A anaerobic enrichment culture, PGM (1/2) = duplicate Postgate enrichment culture

Sample name	Set	MC	CS	DD(kg.m^− 3^)	w_start_	w_final_	Gen1	Gen2	CorrP	Water	30D		R2A_AE1		R2A_AE2		R2A_ANA1		R2A_ANA2		PGM1		PGM2	
							Cq	Cq	Cq	Cq	LD	Cq	LD	Cq	LD	Cq	LD	Cq	LD	Cq	LD	Cq	LD	Cq
cBCV_C20_6m_con	A	Y	Y	1600	0.2	0.226	-	-	+	+	-	+	+	+	+	+	+	+	+	+	+	+	+	+
cBCV_C21_9m_con	A	Y	Y	1600	0.2	0.245	-	n/a	-	+	+	+	+	+	+	+	+	+	+	+	+	+	+	+
cBCV_C22_12m_con	A	Y	Y	1600	0.2	0.238	+	-	n/a	+	+	+	+	+	+	+	+	+	+	+	+	+	+	+
cBCV_C23_18m_con	A	Y	Y	1600	0.2	0.239	+	n/a	+	n/a	+	+	+	+	+	+	+	+	+	+	+	+	+	+
cBCV_C12_9m_T90_IR	A	Y	Y	1600	0.2	0.209	-	n/a	-	n/a	-	-	-	-	-	+	-	-	-	-	-	+	+	+
cBCV_C14_12m_T90_IR	A	Y	Y	1600	0.2	0.232	-	-	n/a	n/a	-	-	-	-	-	-	-	-	-	-	-	-	-	-
cBCV_C16_18m_T90_IR	A	Y	Y	1600	0.2	0.183	-	n/a	-	n/a	-	-	-	-	+	+	-	+	-	-	-	-	-	-
cBCV_C13_9m_T90	A	Y	Y	1600	0.2	0.246	-	-	-	n/a	-	-	-	-	-	-	-	-	-	-	-	-	-	-
cBCV_C15_12m_T90	A	Y	Y	1600	0.2	0.239	-	-	n/a	n/a	-	-	-	-	-	-	-	-	-	-	-	-	-	-
cBCV_C17_18m_T90	A	Y	Y	1600	0.2	0.218	-	n/a	-	n/a	-	-	-	-	-	-	-	-	-	-	-	-	-	-
cBCV_C1_6m_T150_IR	A	Y	Y	1600	0.15	0.211	-	-	-	n/a	-	-	-	-	-	-	-	-	-	-	-	-	-	-
cBCV_C3_9m_T150_IR	A	Y	Y	1600	0.15	0.251	-	n/a	-	n/a	-	-	-	-	-	-	+	+	-	-	-	-	-	-
cBCV_C5_12m_T150_IR	A	Y	Y	1600	0.15	0.205	-	-	n/a	n/a	-	-	-	-	-	-	-	-	-	-	-	-	-	-
cBCV_C7_18m_T150_IR	A	Y	Y	1600	0.15	0.226	-	n/a	-	n/a	-	-	-	-	-	-	-	-	-	-	-	-	-	-
cBCV_C2_6m_T150	A	Y	Y	1600	0.15	0.213	-	n/a	-	n/a	-	-	-	-	-	-	-	-	-	-	-	-	-	-
cBCV_C4_9m_T150	A	Y	Y	1600	0.15	0.22	-	n/a	-	n/a	-	-	+	+	-	-	-	-	-	-	+	+	-	-
cBCV_C6_12m_T150	A	Y	Y	1600	0.15	0.093	-	-	n/a	n/a	-	-	-	-	-	-	-	-	+	+	+	+	-	-
cBCV_C8_18m_T150	A	Y	Y	1600	0.15	0.223	-	n/a	-	n/a	-	-	-	-	-	-	-	-	-	-	-	-	-	-
cMX-80_C11_18m_con	A	Y	Y	1600	0.15	0.196	-	n/a	+	n/a	-	-	+	+	+	+	+	+	+	+	+	+	+	+
cMX-80_C18_18m_T90_IR	A	Y	Y	1600	0.2	0.206	-	n/a	-	n/a	-	-	-	-	-	-	-	-	-	-	-	+	-	-
cMX-80_C19_18m_T90	A	Y	Y	1600	0.2	0.204	-	n/a	-	n/a	-	-	-	-	-	-	-	-	-	-	-	-	-	-
cMX-80_C9_18m_T150_IR	A	Y	Y	1600	0.15	0.166	-	n/a	-	n/a	-	-	-	-	-	-	-	-	-	-	-	-	-	-
cMX-80_C10_18m_T150	A	Y	Y	1600	0.15	0.2	-	n/a	-	n/a	-	-	-	-	-	-	-	-	-	-	-	-	-	-
cBCV_C33_12m_con	B	N	N	1600	0.15	0.118	+	-	n/a	n/a	+	+	+	+	+	+	+	+	+	+	+	+	+	+
cBCV_C32_12m + 6_con	B	N	N + R	1600	0.15	0.243	-	-	n/a	+	+	+	+	+	+	+	+	+	+	+	+	+	+	+
cBCV_C29_12m_T90_IR	B	N	N	1600	0.15	0.125	-	-	n/a	n/a	-	-	-	-	-	-	-	-	-	-	-	-	-	-
cBCV_C28_12 + 6m_T90_IR	B	N	N + R	1600	0.15	0.243	-	-	n/a	-	-	-	-	-	-	-	-	+	-	-	-	-	-	-
cBCV_C31_12m_T90	B	N	N	1600	0.15	0.141	-	-	n/a	n/a	-	-	-	-	-	-	-	-	-	-	-	-	-	-
cBCV_C30_12m + 6_T90	B	N	N + R	1600	0.15	0.242	-	-	n/a	-	-	-	-	-	-	-	-	-	-	-	-	-	-	-
cBCV_C25_12m_T150_IR	B	N	N	1600	0.15	0.101	-	-	n/a	n/a	-	-	-	-	-	-	-	-	-	-	-	-	-	-
cBCV_C24_12m + 6_T150_IR	B	N	N + R	1600	0.15	0.237	-	-	n/a	-	-	-	-	-	-	-	-	-	-	-	-	-	-	-
cBCV_C27_12m_T150	B	N	N	1600	0.15	0.099	-	-	n/a	n/a	-	-	-	-	-	-	-	-	-	-	-	-	-	-
cBCV_C26_12m + 6_T150	B	N	N + R	1600	0.15	0.232	-	-	n/a	-	-	-	-	-	-	-	-	-	-	-	-	-	-	-
cMX-80_C36_12m_con	B	N	N	1600	0.15	0.132	-	-	n/a	n/a	+	+	+	+	+	+	+	+	+	+	+	+	+	+
cMX-80_C34_12m_T90_IR	B	N	N	1600	0.15	0.125	-	-	n/a	n/a	-	-	-	-	-	-	-	-	-	-	-	-	-	-
cMX-80_C35_12m_T90	B	N	N	1600	0.15	0.125	-	-	n/a	n/a	-	-	-	-	-	-	-	-	-	-	-	-	-	-
sBCV_C45_12m_con	C	N	n/a	S	1:3	n/a	+	n/a	n/a	n/a	n	n/a	+	+	+	+	+	+	+	+	+	+	+	+
sBCV_C39_12m_T90_IR	C	N	n/a	S	1:3	n/a	-	n/a	n/a	n/a	-	-	-	-	-	-	-	-	-	-	-	-	-	-
sBCV_C40_12m_T90	C	N	n/a	S	1:3	n/a	-	n/a	n/a	n/a	-	-	-	-	-	-	-	-	-	-	-	-	-	-
sBCV_C37_1m_T150_IR	C	N	n/a	S	1:3	n/a	-	n/a	n/a	n/a	-	-	-	-	-	-	-	-	-	-	-	-	-	-
sBCV_C38_1m_T150	C	N	n/a	S	1:3	n/a	-	n/a	n/a	n/a	-	-	-	-	-	-	-	-	-	-	-	-	-	-
pBCV_C46_12m_con	C	N	n/a	P	0.114	n/a	-	n/a	n/a	n/a	+	+	+	+	+	+	+	+	+	+	+	-	+	+
pBCV_C43_12m_T90_IR	C	N	n/a	P	0.114	n/a	-	n/a	n/a	n/a	-	-	-	-	-	-	-	-	-	-	-	-	-	-
pBCV_C44_12m_T90	C	N	n/a	P	0.114	n/a	-	n/a	n/a	n/a	-	-	-	-	-	-	-	-	-	-	-	-	-	-
pBCV_C41_12m_T150_IR	C	N	n/a	P	0.114	n/a	-	n/a	n/a	n/a	-	-	-	-	-	-	-	-	-	-	-	-	-	-
pBCV_C42_12m_T150	C	N	n/a	P	1:6	n/a	-	n/a	n/a	n/a	-	-	-	-	+	+	-	-	-	-	-	-	-	-
sMX-80_C55_12m_con	C	N	n/a	S	1:6	n/a	+	n/a	n/a	n/a	n/a	n/a	+	+	-	-	+	+	+	+	+	+	+	+
sMX-80_C49_12m_T90_IR	C	N	n/a	S	1:6	n/a	-	n/a	n/a	n/a	-	-	-	-	-	-	-	-	-	-	-	-	-	-
sMX-80_C50_12m_T90	C	N	n/a	S	1:6	n/a	-	n/a	n/a	n/a	-	-	-	-	-	-	-	-	-	-	-	-	-	-
sMX-80_C47_1m_T150_IR	C	N	n/a	S	1:6	n/a	-	n/a	n/a	n/a	-	-	-	-	-	-	-	-	-	-	-	-	-	-
sMX-80_C48_1m_T150	C	N	n/a	S	1:6	n/a	-	n/a	n/a	n/a	-	-	-	-	-	-	-	-	-	-	-	-	-	-
pMX-80_C56_12m_con	C	N	n/a	P	0.074	n/a	-	n/a	n/a	n/a	+	+	+	+	+	+	+	+	+	+	+	+	+	+
pMX-80_C53_12m_T90_IR	C	N	n/a	P	0.074	n/a	-	n/a	n/a	n/a	-	-	-	-	-	-	-	-	-	-	-	-	-	-
pMX-80_C54_12m_T90	C	N	n/a	P	0.074	n/a	-	n/a	n/a	n/a	-	-	-	-	-	-	-	-	-	n/a	-	-	-	-
pMX-80_C51_12m_T150_IR	C	N	n/a	P	0.074	n/a	-	n/a	n/a	n/a	-	-	-	-	-	-	-	-	-	-	-	-	-	-
pMX-80_C52_12m_T150	C	N	n/a	P	0.074	n/a	-	n/a	n/a	n/a	-	-	-	-	-	-	-	-	-	-	-	-	-	-

**Fig. 2:**
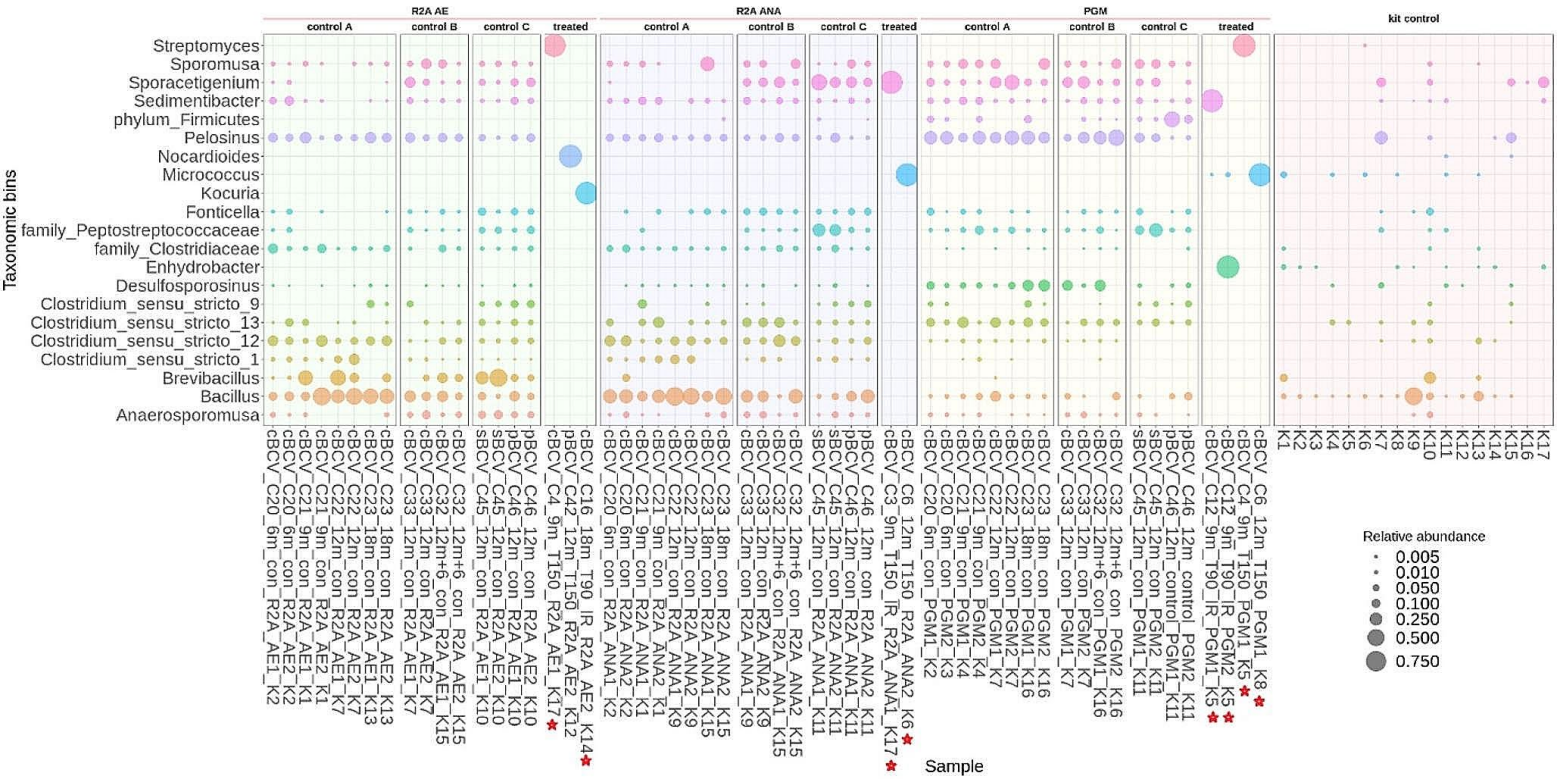
Microbial community profiles (ASV grouped at genus level) for positive enrichment cultures from BCV samples in the long-term experiment. Only those genera ≥ 0.5% relative abundance are shown. Sample names indicate treatment and bentonite type: cBCV/pBCV/sBCV = compacted/ powder/ suspension samples, C = sample number, 6–18 m = exposure duration, T = temperature (90/150), IR = irradiated samples, con = control samples. AE (1/2) = R2A aerobic medium, ANA (1/2) = R2A anaerobic medium, PGM (1/2) = Postgate medium. K = co-isolated kit controls (also listed at the end of each sample name). Red stars denote the culture samples from treated samples of experimental set A dismantled semi-sterilely

These positive BCV cultures from treated samples were mainly dominated by a single genus, such as *Kocuria*, *Enhydrobacter*, *Micrococcus*, *Nocardioides*, *Sedimentibacter*, *Streptomyces* or *Sporacetigenium* (Fig. 2). Noteworthy, different species (*Sedimentibacter* and *Enhydrobacter*, respectively) dominated positive duplicate PGM culture samples in the case of set A sample C12 (90 °C, IR, 9 m incubation). Only one positive culture sample was detected with MX-80 bentonite (Table 1); however, due to technical issues, sequencing was not performed on this sample.

In comparison, almost all BCV and MX-80 control samples from sets A, B and C from the long-term experiment resulted in positive cultures with diverse microbial compositions (Table 1). In BCV samples (all three sets), the R2A (aerobic and anaerobic) medium exhibited common bentonite genera belonging to the order Bacillales, family Clostridiaceae, and genus *Pelosinus*. In addition, some rare genera were also identified, including *Sedimentibacter*, *Sporomusa*, *Sporacetigenium*, *Fonticella*, and *Anaerosporomusa* (Fig. 2). In PGM cultivations from controls to set A, B and C, genera such as *Pelosinus* and *Sporacetigenium* were most abundant, followed by the genus *Desulfosporosinus*, *Bacillus*, a range of *Clostridium* spp. and genera belonging to family Peptostreptococcaceae along with rarer genera such as *Sporomusa* and *Anaerosporomusa* (Fig. 2).

**Fig. 3:**
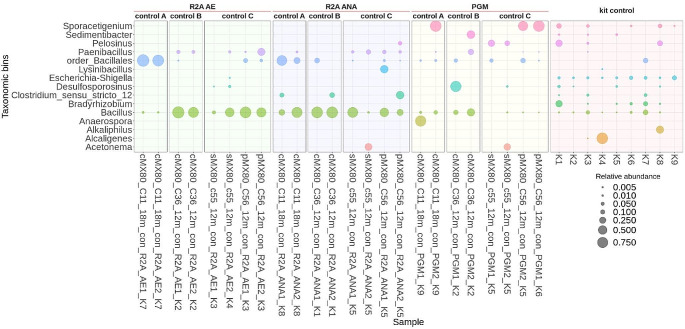
Microbial community profiles (ASV grouped at genus level) for positive enrichment cultures from MX-80 samples in the long-term experiment. Only those genera ≥ 0.5% relative abundance are shown. Sample names indicate treatment and bentonite type (MX-80 bentonite): cMX-80/p MX-80/s MX-80 = compacted/powder/suspension samples, C = sample number, 12–18 m = exposure duration, con = control samples. AE (1/2) = R2A aerobic medium, ANA (1/2) = R2A anaerobic medium, PGM (1/2) = Postgate medium. K = co-isolated kit controls (also listed at the end of each sample name)

In control MX-80 samples, the microbial composition was very similar across all three sets for each enrichment culture type. The R2A enrichments (aerobic and anaerobic) were mainly dominated by genera belonging to members of the order Bacillales, together with the genus *Paenibacillus*, while the genera *Clostridium* and *Lysinibacillus* were also recorded in R2A anaerobic cultivations, in the case of PGM, genera such as *Sporacetigenium*, *Desulfosporosinus*, *Anaerospora*, *Sedimentibacter*, and *Pelosinus*, or members of the order Bacillales and rare genera *Acetonema* tended to dominate (Fig. 3).

#### Bentonite

Relative quantification of 16S rRNA gene copies in fresh BCV and MX-80 bentonite samples from the long-term experiment (A, B, and C) revealed no noticeable growth in total microbial biomass (indicated by 16S rRNA gene copy numbers) in any of the fresh samples subjected to continuous heating or a combination of heat and irradiation (Table 1). The same result was also obtained for the 30D natural incubations of these samples. None of the incubated suspensions proved positive using either qPCR or microscopy (cell extraction) observations (Table 1). Furthermore, no microbial proliferation was observed in treated compacted bentonite samples from set B, even after 6-months saturation at laboratory temperature after the heat treatment or after a further 30D natural incubation in suspended form. Similarly, the water used for long-term saturation showed no signs of microbiological growth (Table 1). In accordance with the qPCR/microscopic results, microbial compositions detected in all treated bentonite samples were similar to those of the co-extracted kit controls, verifying the absence of a true genetic signal (Supplementary Figures S1 and S2 (set A), S3 and S4 (set B), and S5 and S6 (set C)). Because of the overall lack of microbial proliferation in all treated bentonite samples, no difference could be detected between experimental sets at either temperature (90 °C and 150 °C) or with/without irradiation.

Some fresh BCV and MX-80 control samples showed signs of microbial proliferation based on 16S rRNA qPCR. Positive Cq values were detected in compacted bentonite samples after at least 12-months exposure in sets A and B controls and in 12-months incubated suspension samples in set C. The 30D natural incubations of the control samples were positive in all cases, as were the water samples from the saturation reservoirs (Table 1). The bentonite corrosion layer samples from the BCV controls were only positive after 6- and 18-months incubation, and those from the MX-80 controls after 18-months, in sets A and B.

**Fig. 4:**
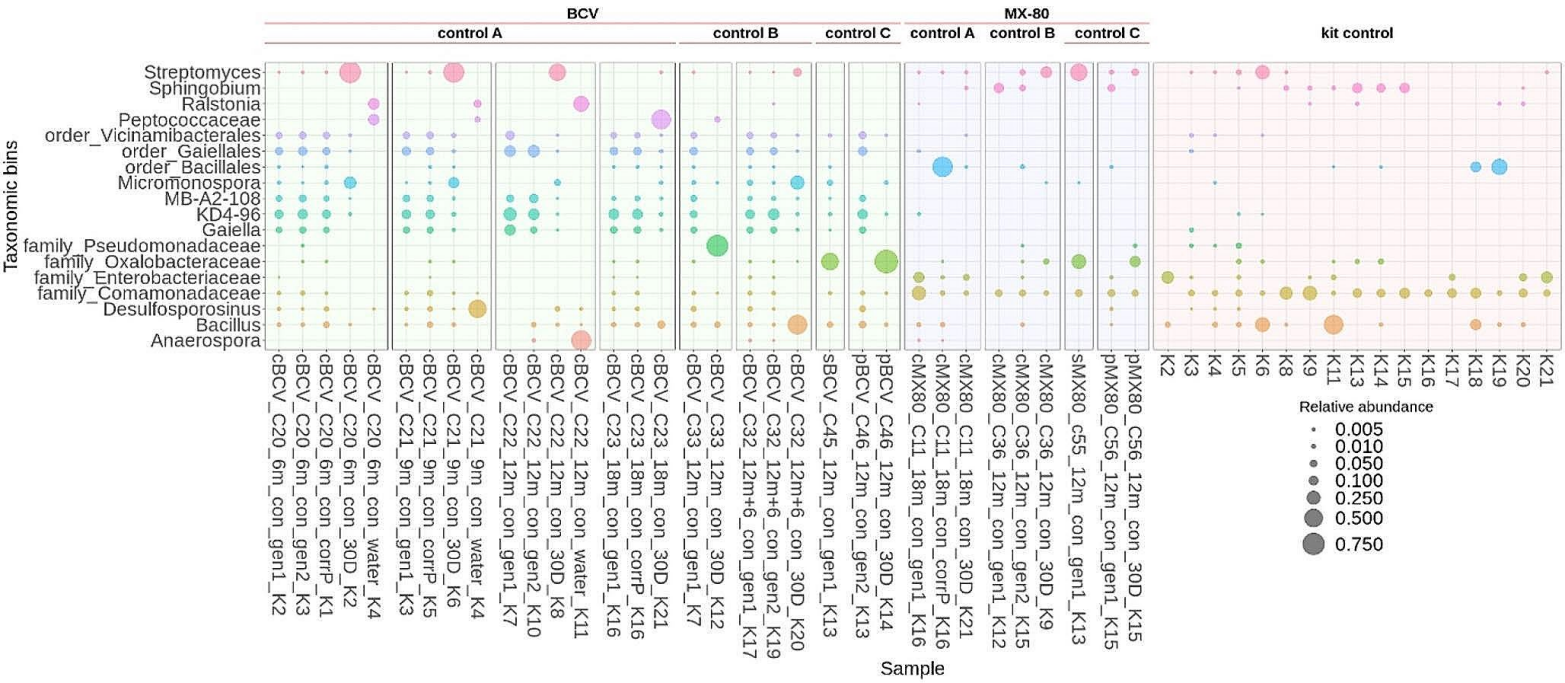
Microbial community profiles (ASV grouped at genus level) for control bentonite samples from the long-term experiment. Only genera ≥ 0.5% relative abundance are shown. Sample names indicate the treatment and bentonite type (BCV/MX-80): c/p/s = compacted/ powder/ suspension samples, C = sample number, 6–18 m = exposure duration, con = control samples, Gen (1/2) = fresh bentonite samples, 30D = 30-day naturally incubated sample in suspended form, CorrP = corrosion layer, water = reservoir water. K = co-isolated kit controls (also listed at the end of each sample name)

NGS analysis revealed that most of the control compacted bentonite samples shared a similar microbial composition pattern regardless of the experimental set (Fig. 4). In BCV samples, members of the orders Vicinamibacterales, Gaiellales, or Bacilales were detected, together with the taxa MB-A2-108 and KD4-96. On the other hand, a very weak signal was detected in compacted MX-80 samples, with the pattern very similar to the kit controls. In 12-months incubated control bentonite suspensions, members of the family Oxalobacteraceae dominated in the BCV suspension and members of the family Oxalobacteraceae and genus *Streptomyces* in the MX-80 suspension. In 30D naturally incubated BCV control samples, the genera *Streptomyces*, *Micromonospora*, and *Bacillus*, or members of the families Oxalobacteraceae and Peptococcaceae, dominated, while the genera *Pseudomonas* or *Streptomyces* dominated in 30D naturally incubated MX-80 control samples. The genera *Desulfosporosinus*, *Anaerospora*, and *Ralstonia* dominated the water used for sample saturation. In the case of BCV, community composition in the control sample corrosion layers was similar to that in the other bentonite samples, and the proliferation of any particular genus could not be clearly distinguished. In the corrosion layer of MX-80 sample, only one non-specified member of the order Bacillales was enriched. However, this particular taxon is also frequently detected in the kit controls and thus might represent contaminant species (Fig. 4).

For BCV samples, ANOVA indicated the most significant difference between treated and control samples (*p* < 0.001), explaining over 14 % of the variability in the data (see Supplementary Table S8). Sample type (fresh sample/30D/CorrP) and experimental set (A, B, or C) were also significant factors (*p* < 0.001 and < 0.01, respectively), explaining a further 3 and 6 % of variability, respectively. In the case of MX-80, the most significant difference was between experimental sets (*p* < 0.001), which explained over 17% of the variability in the data. Likewise, treatment (treated/control) and sample type (fresh sample/30D/CorrP) also caused statistically significant differences in microbial composition (*p* < 0.001 and < 0.01, respectively), explaining a further 6.6 and 7 % variability, respectively. No effect of irradiation was observed on microbial composition in either BCV or MX-80 bentonite samples.

**Fig. 5:**
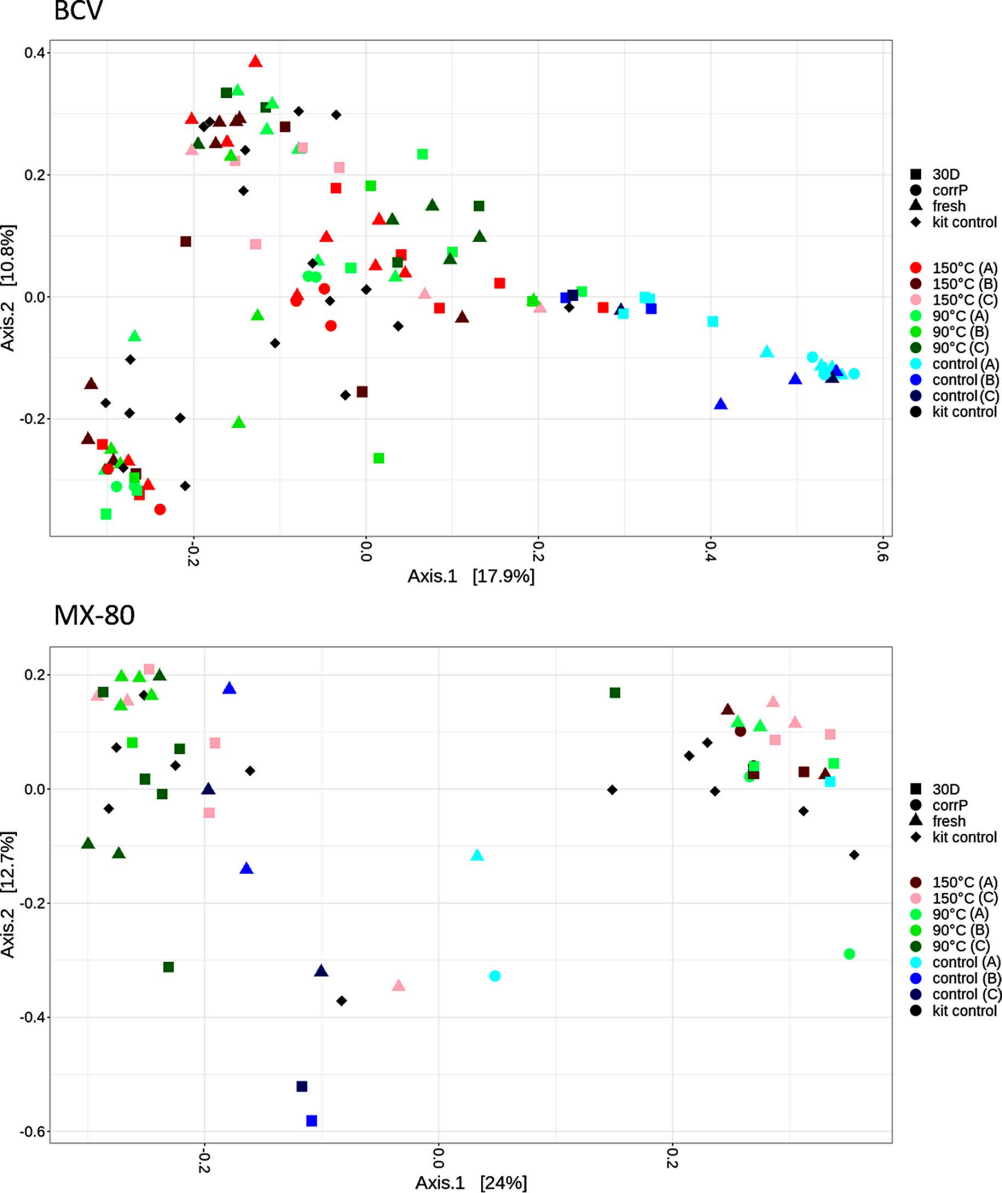
Principal-coordinate analysis (PcoA) ordination plot based on Bray-Curtis distance metrics, showing the grouping of BCV (above) and MX-80 (below) bentonite samples from the long-term experiment based on their microbial composition. 30D = 30-day naturally incubated sample in suspended form, CorrP = corrosion layer, fresh = fresh bentonite samples, kit control = co-isolated kit controls, control = untreated control samples, 90 °C/150°C = temperature applied, A,B + C = experimental set

The graph visualizing PCoA results showed that most of the BCV control samples (in blue, Fig. 5) formed a distinct cluster, while the treated samples (in red and green, Fig. 5) tended to group together with the kit control samples (in black, Fig. 5). Such a pattern implies a lack of genetic signal in most of the treated samples. An effect of the experimental set or sample type on sample distribution was only clearly visible in the case of BCV control samples, where the 30D naturally incubated samples (blue squares, Fig. 5) and fresh samples (blue triangles, Fig. 5) tended to form separate sub-clusters. On the other hand, treated MX-80 samples tended to cluster based on the experimental set and sample type. However, these samples, similarly to BCV, clustered together with the kit controls, implying a lack of valid genetic signal in the MX-80 treated samples. The PCoA further showed only a very weak difference between control and treated MX-80 samples, which again well corresponds to the overall weaker genetic signal of MX-80 samples compared to the BCV samples.

### Additional experiment

#### Cultivations

In the additional experiment with heated BCV powder, microbial proliferation was detected in several culture samples, especially in samples exposed to 90 °C for 1–6 months. In comparison, samples exposed to 150 °C only exhibited microbial proliferation in one culture sample at 3 months (Table 2).

**Table 2:** Summary result table - qPCR (cq values) and microscopy (L/D) results obtained from the additional experiment with BCV bentonite. Sample names indicate treatment: p = powder samples, 1–6 m = exposure duration, T90/150 = applied temperature, Gen = fresh bentonite samples for genetics, 30D = 30-day naturally incubated suspension samples, R2A_AE (1/2) = duplicate R2A aerobic enrichment culture, R2A_ANA (1/2) = duplicate R2A anaerobic enrichment culture, PGM (1/2) = duplicate postgate enrichment culture

Sample name	Type	Gen	30D		R2A_AE1		R2A_AE2		R2A_ANA1		R2A_ANA2		PGM1		PGM2	
		Cq	L/D	Cq	L/D	Cq	L/D	Cq	L/D	Cq	L/D	Cq	L/D	Cq	L/D	Cq
pBCV_1_1m_T90	Powder	-	-	-	+	+	+	+	+	+	+	+	+	+	+	+
pBCV_2_1m_T90	Powder	-	-	-	-	-	-	-	-	-	-	-	-	-	-	-
pBCV_1_3m_T90	Powder	-	-	-	+	+	-	-	-	-	-	-	-	-	-	-
pBCV_2_3m_T90	Powder	-	-	+	-	-	-	-	-	+	-	-	-	-	-	-
pBCV_1_6m_T90	Powder	-	-	-	-	-	-	-	-	-	-	-	-	-	-	-
pBCV_2_6m_T90	Powder	-	-	-	-	-	-	-	-	-	-	-	-	+	-	+
pBCV_1_1m_T150	Powder	-	-	-	-	-	-	-	-	-	-	-	-	-	-	-
pBCV_2_1m_T150	Powder	-	-	-	-	-	-	-	-	-	-	-	-	-	-	-
pBCV_1_3m_T150	Powder	-	-	-	-	-	-	-	-	-	-	-	+	+	-	-
pBCV_2_3m_T150	Powder	-	-	-	-	-	-	-	-	-	-	-	-	-	-	-
pBCV_1_6m_T150	Powder	-	-	-	-	-	-	-	-	-	-	-	-	-	-	-
pBCV_2_6m_T150	Powder	-	-	-	-	-	-	-	-	-	-	-	-	-	-	-

**Fig. 6:**
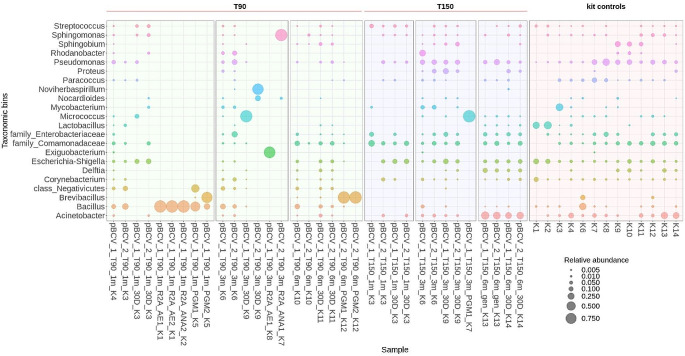
Microbial community profiles (ASV grouped at genus level) for BCV bentonite samples from the additional experiment. Only those genera ≥ 0.5% relative abundance are shown. Sample names indicate treatment: P = powder samples, 1–6 m = exposure duration, T90/150 = temperature applied, 30D = 30-day naturally incubated sample in suspended form, AE (1/2) = R2A aerobic medium, ANA (1/2) = R2A anaerobic medium, PGM (1/2) = Postgate medium. K = co-isolated kit controls (also listed at the end of each sample name)

In positive culture samples, the genus *Bacillus* was dominant in both R2A (both aerobic and anaerobic) and the PGM medium, the latter also exhibiting the presence of the genus *Brevibacillus* and class Negativicutes as significant communities after 1-month exposure to a thermal load of 90 °C. Interestingly, a shift in microbial composition became apparent after 3-months of exposure to the same thermal conditions, with different genera, such as *Exiguobacterium* (R2A aerobic) or *Sphingomonas* (R2A anaerobic), dominating particular media. The genus *Brevibacillus* was detected in PGM medium after 6-months exposure to 90 °C; however, in samples heated to 150 °C, only one positive culture sample was detected, dominated by the genus *Micrococcus* (Fig. 6).

#### Bentonite

Corresponding to the culture results, almost none of the heated BCV samples or their subsequent 30D natural incubations in additional experiments showed signs of microbial proliferation, as indicated by the similarity between microbial compositions detected in all bentonite samples. The patterns noted were also very similar to those from the extraction kit controls, further indicating a lack of true signal in these samples, as in the long-term experiment (Fig. 6). The only exception was BCV samples heated at 90 °C for 3 months, where positive qPCR values and proliferation of genus *Micrococcus* and *Noviherbaspirillum* were detected (Fig. 6).

## Discussion

In this study, we aimed to simulate bentonite conditions in the proximity of radioactive waste canisters during the DGR hot phase to assess the response of indigenous bentonite microorganisms, with a specific focus on their ability to regenerate after such extreme conditions have passed. In doing so, we examined the impact of multiple repository-relevant stressors, i.e. high temperatures (90–150 °C) and the combined effect of high temperatures plus irradiation from a ^60^Co source. In addition, the water content and bentonite/water/air ratio (compacted/non-compacted) were varied to assess their effect on microbial survivability. Multiple time points were also included to estimate the effect of exposure duration.

Stroes-Gascoyne and West (1997) demonstrated that the combined impact of heat and irradiation elicited a biphasic survival response, allowing for the coexistence of two distinct microbial populations with varying tolerance mechanisms. In this case, one population exhibited decreased radiation resistance with an increase in temperature, while the other showed increased resistance to irradiation with an increase in temperature, indicating a synergistic interaction between heat and radiation. Further experiments have demonstrated that radiation resistance in microorganisms could be incidental, being a result of their adaptation to physiological stress (Mattimore and Battista 1996). Our data demonstrated a very strong effect of long-term exposure to both temperatures tested (90 and 150 °C), leading to a dramatic loss in viability and cultivability of indigenous bentonite microorganisms, with similar effects observed in both BCV and MX-80 bentonite. In fact, the effect of temperature was so strong that the effect of other factors, such as irradiation or water availability, could not be evaluated, though these factors are known to influence the viability and survivability of bacteria in bentonite (Stroes-Gascoyne et al. 1995; Motamedi et al. 1996; Brown et al. 2015).

An additional experiment with heated BCV powder enabled us to detect the effect of exposure duration. We were able to demonstrate that bentonite powder samples heated to 90°C still showed cultivability after 6-months exposure, but that cultivability had decreased dramatically after 12-months exposure. After heating BCV powder to 150 °C, just 1-months exposure resulted in (probably complete) bentonite sterilization. These results contradict the previous data from the EURAD project ‘HITEC’ (Kašpar et al. 2021), where BCV powder was also heated to 150 °C for 6 and 12 months. In this case, possible microbial proliferation was detected after 6-months exposure and possibly also after 12-months exposure. However, these data only relied upon the natural incubation of heated bentonite in its suspended form, and no enrichment cultures or microscopy analyses were performed to confirm the findings independently. Additionally, microbiological analysis was performed on the material as received. Hence, it cannot be ruled out that the growth detected may have resulted from unintentional contamination during sampling or storage post-sampling.

In our study, both enrichment cultures in different media and natural incubations of bentonite suspensions were included to test which cultivation conditions were more suitable for demonstrating effects on microbial survivability. While natural incubations were successfully used in our previous experiments (Kašpar et al. 2021; Bartak et al. 2023) to assess the reaction of bentonite microorganisms to elevated pressure and temperature, the enrichment culture approach was clearly superior to natural incubations for detecting microbial survivability in the present study. This finding agrees well with previous studies, where an excess of available nutrients promoted microbial recovery following harsh treatment, e.g., through irradiation (Nicholson et al. 2000; Ratto and Itavaara 2012; Gilmour et al. 2021). On the other hand, enrichment cultures are more susceptible to contamination as they also represent a suitable environment for many contaminant species, which can be a significant drawback. In our study, the compacted bentonite samples, which were to be shared with another team, had to be dismantled in an anaerobic glove box, where completely sterile conditions could not be maintained despite precautions being taken to minimize this risk. Several positive cultures were detected originating from shared treated samples in set A. On the other hand, only two positive cultures were obtained from sets B and C, where the samples were dismantled in a laminar flow box under controlled sterile conditions. This comparison implies possible contaminations occurring during shared sample processing in the anaerobic glove box, and the culture results from set A thus need to be evaluated with caution. The Gram-positive non-spore-former *Nocardioides* (Lee et al. 2017) was the only genus detected in cultures from BCV samples heated at 150 °C for 12 months in experimental set C (Fig. 2) dismantled under completely sterile conditions, and it was also abundant in 30D natural incubation of BCV powder exposed to the 90 °C for 3 m (Fig. 6). *Nocardioides* was also detected in the compacted BCV bentonite, but at low relative abundance (< 0.1%). Interestingly, the presence of this aerobic genus in bentonite samples could be related to the ability of bentonite to trap oxygen molecules that may be available for aerobic bacteria (Burzan et al. 2022). As for the genera identified in positive enrichment cultures from semi-sterile dismantled samples of experimental set A, a spore-forming genus *Kocuria* was detected in irradiated powder BCV samples heated at 90 °C for 18- months. This genus was also reported in soils contaminated with toxic metals or exposed to high levels of ionizing radiation (Timkina et al. 2022). *Sporacetigenium* detected in irradiated powder BCV samples heated at 150 °C for 9- months is a moderately thermophilic bacterium that can withstand high temperatures by forming spores (Chen et al. 2006; Martinez-Moreno et al. 2024) and was also dominant in cultures from BCV control samples (Fig. 1). Several other genera were further detected in enrichment cultures from the treated samples of set A, such as facultative anaerobic Gram-negative non-spore-former *Enhydrobacter* (Staley et al. 1987) or bacteria from the class Actinomycetes such as gram-positive non-spore forming *Micrococcus* or spore-forming *Streptomyces* (Farda et al. 2022) and *Sedimentibacter* (Yu et al. 2014) that are commonly reported in the soil.

Our study has shown that untreated bentonites (BCV and MX-80) host a diverse range of indigenous microbial communities, many of which are known to be highly resistant and capable of germinating to a metabolically active state once conditions become favorable (Stroes-Gascoyne et al. 2002; Masurat et al. 2010b; Gilmour et al. 2021). In accordance with this, we observed that the cultivability of bentonite bacteria, including SRB, remained unaffected in control bentonite samples, even after compacting to 1600 kg.m^-3^ dry density for 18 months. Compaction to 1600 kg.m^-3^ dry density, therefore, proved incapable of eradicating bentonite microorganisms, which could pose a risk to canister stability in the case of bentonite density decrease. This risk is specifically associated with the presence of sulfate-reducing bacteria, whose activity dependence on the compacted bentonite density has been extensively studied (e.g. Masurat et al. 2010a; Pedersen 2010; Bengtsson and Pedersen 2017; Bengtsson et al. 2017). In accordance with these results, we observed that bentonite compaction did appear to limit ongoing microbial activity in our compacted bentonite samples. Though qPCR analysis indicated a gradual increase in relative microbial abundance in compacted control samples with time and possible ongoing microbial proliferation in corrosion layers near the steel coupons in control samples, the proliferation of specific genera could not be detected by NGS in any of these samples. Thus, our data could not confirm ongoing MIC in the control samples, possibly due to a severe slowdown in all biological and chemical processes in the compacted bentonite due to its high dry density.

It has been predicted that, during the initial hot phase of DGR evolution, when temperature and irradiation reach their peak, prevailing conditions would cause a dramatic loss in microbial survivability, potentially resulting in the creation of abiotic zones around the waste canisters (Stroes-Gascoyne and West 1997; Aoki et al. 2010). The results obtained for our treated samples agree well with this hypothesis, showing that bentonite microbial activity (aerobic and anaerobic heterotrophs and SRB, many of which belong to spore-forming microorganisms), and thus the risk of MIC, is strongly suppressed when the bentonite is long term exposed to the temperature 90 °C or higher, which are the conditions expected near the canister surface (Johnson et al. 2002; Hicks et al. 2009; SKB 2010a). However, the formation and spatial distribution of such a presumable abiotic zone will depend upon overall temperature conditions within the DGR. The presence of highly resistant bentonite microorganisms capable of germinating to a metabolically active state when conditions become favorable was clearly demonstrated in our heated samples (up to 6-months exposure to 90 °C), with some bacteria still showing cultivability in enriched media. Similarly, in our previous short-term experiment with bentonite suspension exposed to temperatures 60–90 °C for 1-month, we demonstrated that microbial activity decreased with temperature, and we identified several thermophilic taxa. A temperature of 90 °C limited microbial activity and proliferation in all bentonite suspensions, which is in good accordance to the current results. However, more data are needed, especially long-term experiments testing the microbial response to nearly limiting temperatures, to assess the possible effect of temperature gradients on microbial survivability in the bentonite sealing layer. A further non-resolved question is the microbial mobility through the compacted bentonite, as this can lead to reinfestation of the abiotic zone from the surrounding environment. Combining microbial survivability data with mathematical modelling of the environmental conditions in DGR across its lifetime has excellent potential for increasing the accuracy of long-term predictions of microbial effects in DGRs.

## Conclusion

Our investigation focused on the impact of repository-relevant stressors on indigenous microorganisms in bentonite. Elevated temperatures (90 °C and 150 °C) significantly reduced the viability of bentonite microorganisms, with consistent effects observed in both BCV and MX-80 bentonites. The effects of additional factors, such as irradiation (repository-relevant dose rate of 0.4 Gy.h^− 1^), bentonite/water/air ratio, and water availability, could not be distinctly assessed due to the strong effect of temperature. The study emphasized the critical role of exposure duration in understanding microbial responses to heat treatment.

Our experiments employed enrichment cultures and natural incubation to assess microbial survivability under different conditions. While natural incubations were successful in previous studies, our current findings favor the enrichment culture approach despite susceptibility to contamination. Controlled sterile conditions during sample processing will be necessary to mitigate contamination issues.

The study indicated little potential for microbially-induced corrosion to waste canisters during the initial hot phase of DGR evolution, mostly due to the detrimental impact of high temperatures on microbial viability. Our data support the hypothesis of the evolution of an abiotic zone in bentonite surrounding canisters during the early repository stages, which will be contingent upon overall temperature and water saturation conditions within the DGR. Further long-term experiments and mathematical modeling are recommended to refine predictions of microbial effects over time in the repository.

### Electronic supplementary material

Below is the link to the electronic supplementary material.

Supplementary Material 1

Supplementary Material 2

## Data Availability

Sequencing data are openly available in the NCBI database (BioProject ID: PRJNA1086824).
