# Supplementary material for: Dramatic loss of microbial viability in bentonite exposed to heat and gamma radiation: implications for deep geological repository
Source: World J Microbiol Biotechnol. 2024 Jul 11;40(9):264. doi: 10.1007/s11274-024-04069-w (PMC11239606; doi:10.1007/s11274-024-04069-w)
Supplement: Supplementary file 2 — Supplementary Material 2 [file 11274_2024_4069_MOESM2_ESM.docx]

**Dramatic loss of microbial viability in bentonite exposed to heat and gamma radiation: Implications for deep geological repository**

Deepa Bartak^1^, Šárka Šachlová^2^, Vlastislav Kašpar^2^, Jakub Říha^1^, David Dobrev^2^, Petr Večerník^2^, Veronika Hlaváčková^1^, Michaela Matulová^3^ and Kateřina Černá^1^*

^1^ Institute for Nanomaterials, Advanced Technologies and Innovation, Technical University of Liberec, Bendlova 7, 460 01 Liberec, Czech Republic

^2^ Disposal processes and safety, ÚJV Řež, a. s., Hlavní 130, 250 68 Husinec, Czech Republic

*Corresponding author


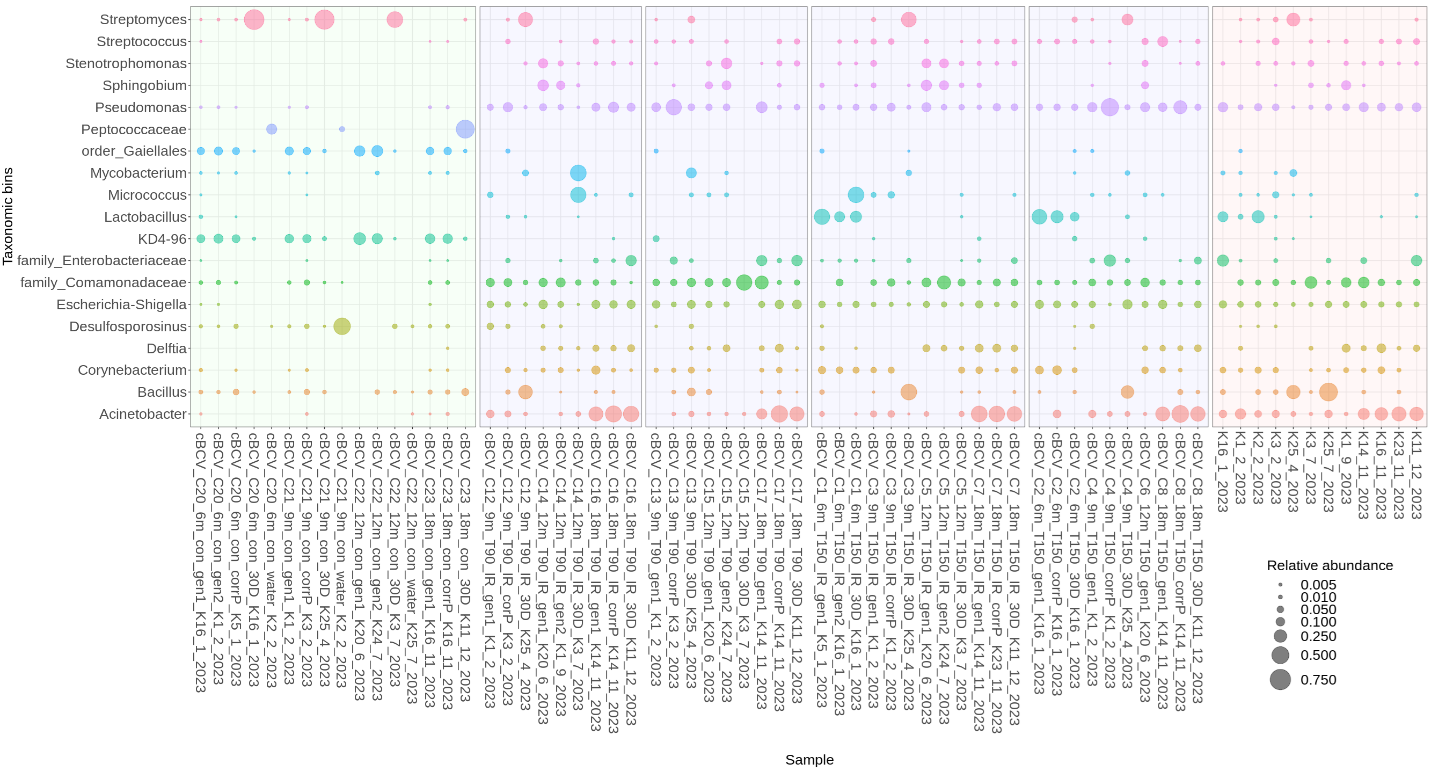
Figure S1: Microbial composition (ASV grouped at genus level) of BCV bentonite samples (experimental set A) from the long-term experiment. Only genera at or above 0.5% relative abundance are shown. Sample names indicate the treatment and bentonite type: cBCV - compacted samples, C - sample number, 6-18m - exposure duration, T - temperature (90/150 °C), IR - irradiated samples, con - control samples, gen1/2 = fresh bentonite samples, 30D = 30-days naturally incubated sample in suspended form, CorrP = corrosion layer, water = reservoir water. K - co-isolated kit controls (also listed at the end of each sample name).


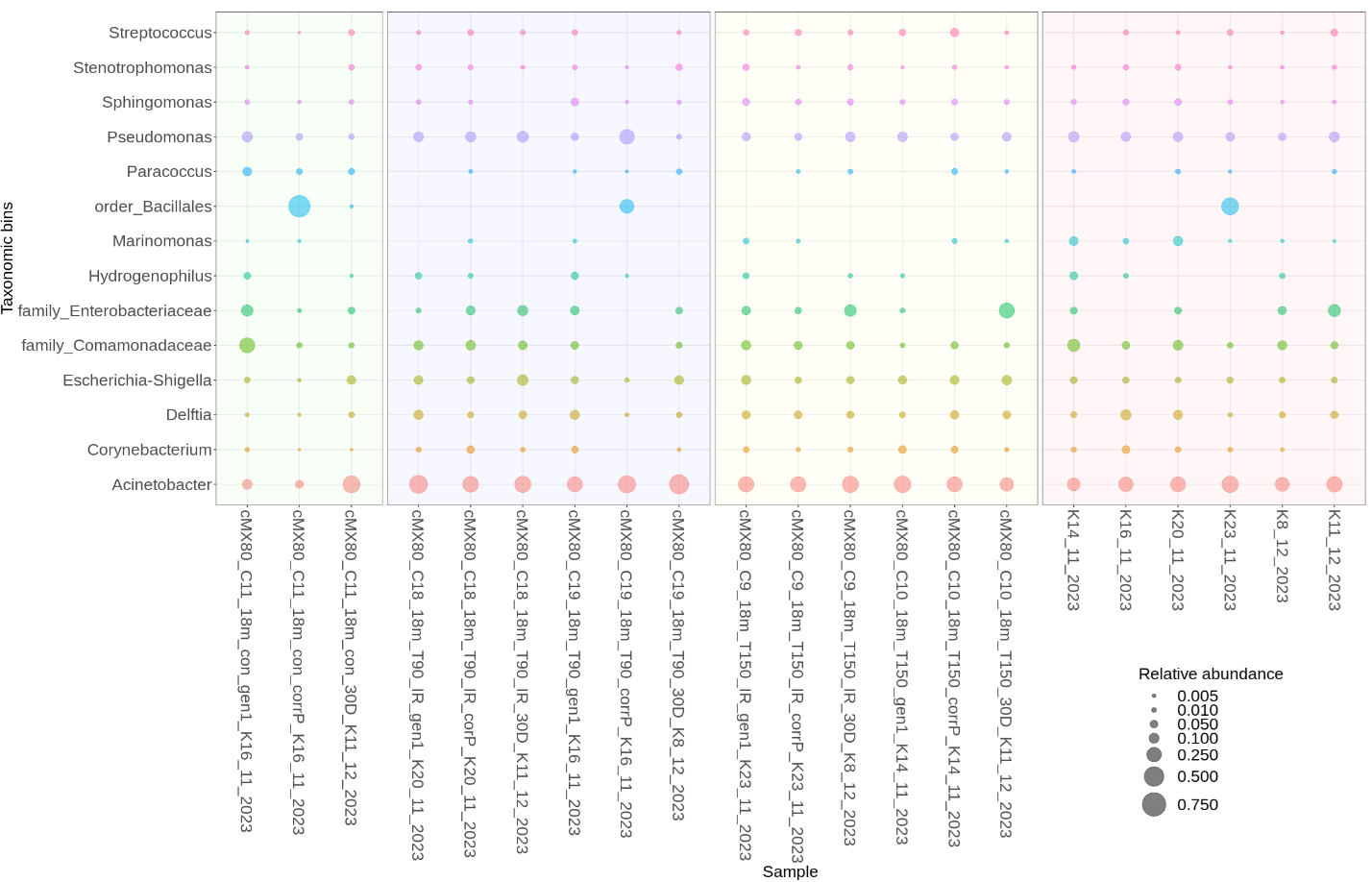


Figure S2: Microbial composition (ASV grouped at genus level) of MX-80 samples (experimental set A) from the long-term experiment. Only genera at or above 0.5% relative abundance are shown. Sample names indicate the treatment and bentonite type: cMX-80 - compacted samples, C - sample number, 6-18m - exposure duration, T - temperature (90/150), IR - irradiated samples, con - control samples, gen1 = fresh bentonite samples, 30D = 30-days naturally incubated sample in suspended form, CorrP = corrosion layer, K - co-isolated kit controls (also listed at the end of each sample name).


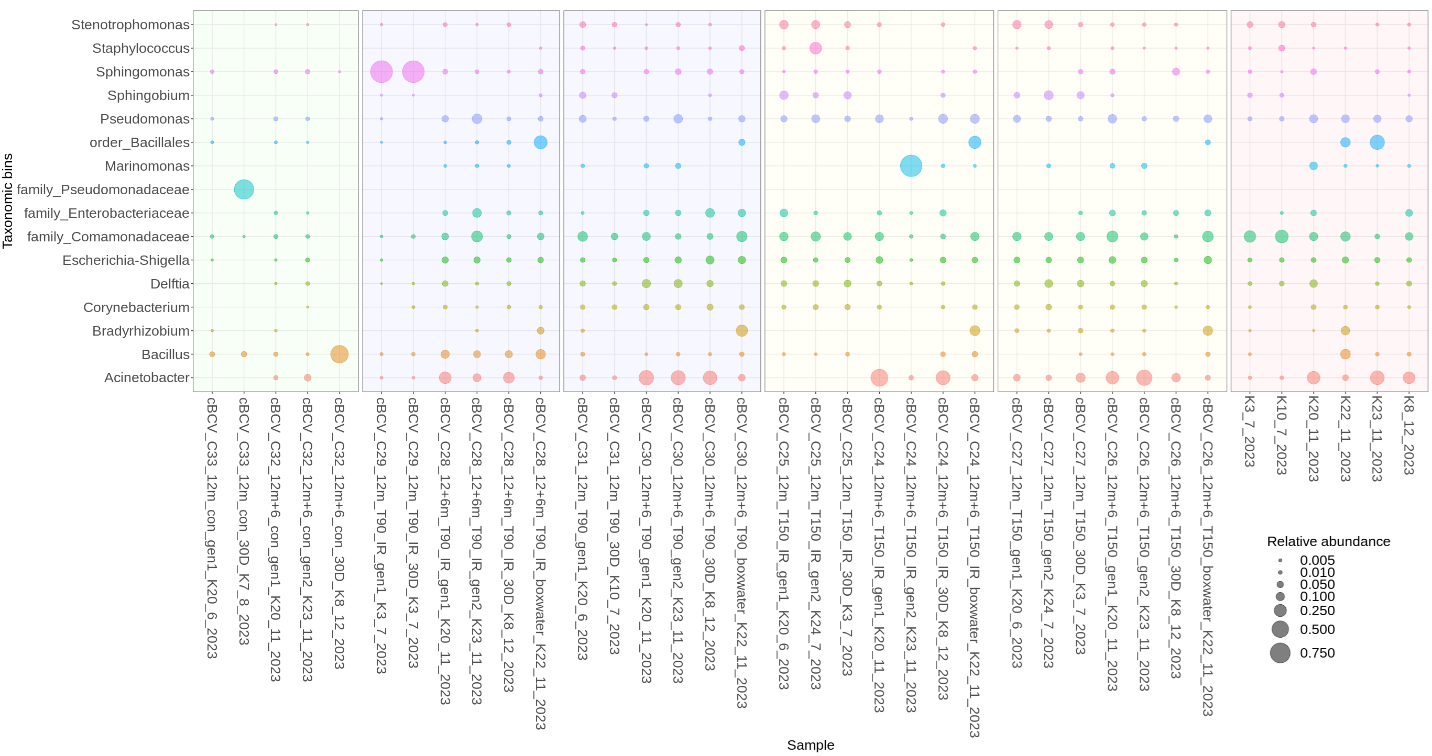


Figure S3: Microbial community composition (ASV grouped at genus level) of BCV samples (experimental set B) from the long-term experiment. Only genera at or above 0.5% relative abundance are shown. Sample names indicate the treatment and bentonite type: cBCV - compacted samples, C - sample number, 6-18m - exposure duration, T - temperature (90/150), IR - irradiated samples, con - control samples, gen1/2 = fresh bentonite samples, 30D = 30-days naturally incubated sample in suspended form, boxwater = reservoir water and K - co-isolated kit controls (also listed at the end of each sample name).


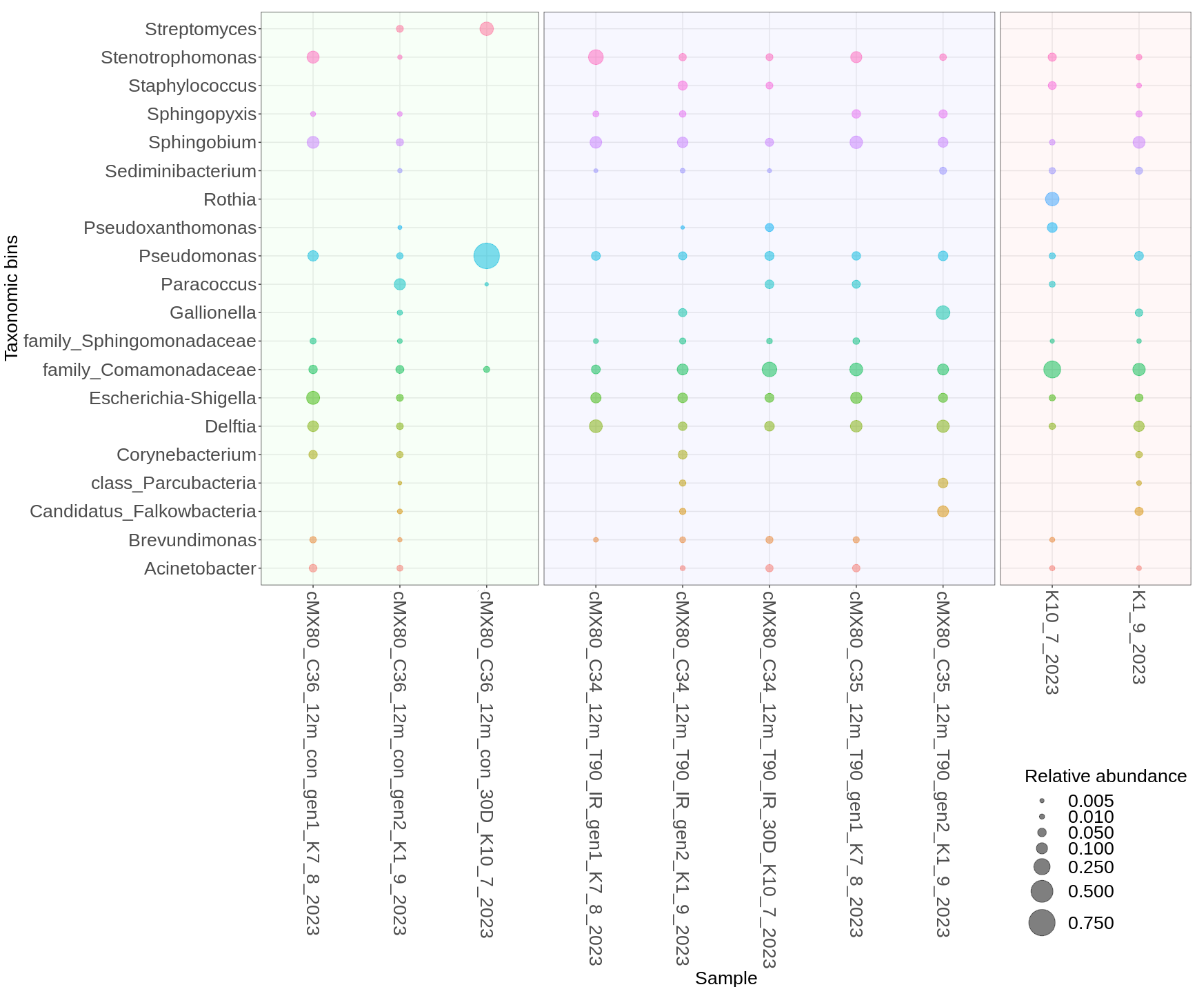


Figure S4: Microbial composition (ASV grouped at genus level) of MX-80 samples (experimental set B) of the long-term experiment. Only genera at or above 0.5% relative abundance are shown. Sample names indicate the treatment and bentonite type: cMX-80 - compacted samples, C - sample number, 12m - exposure duration, T - temperature (90/150), IR - irradiated samples, con - control samples, gen1/2 = fresh bentonite samples, 30D = 30-days naturally incubated sample in suspended form and K - co-isolated kit controls (also listed at the end of each sample name).


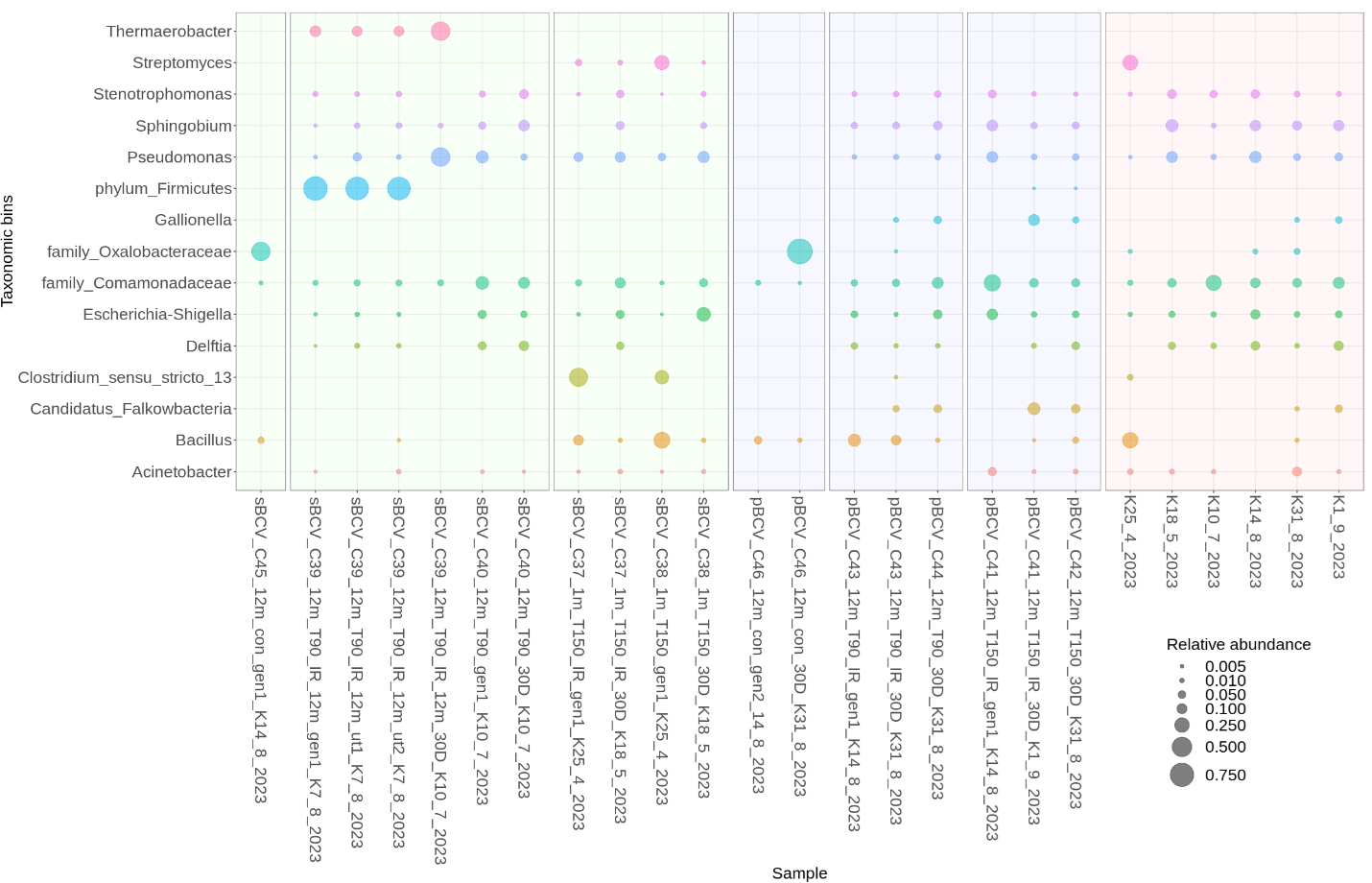


Figure S5: Microbial composition (ASV grouped at genus level) of BCV samples (experimental set C) from the long-term experiment. Only genera at or above 0.5% relative abundance are shown. Sample names indicate the treatment and bentonite type: sBCV/ pBCV – suspension/ powder samples, C - sample number, 1 - 12m - exposure duration in months, T - temperature (90/150), IR - irradiated samples, con - control samples, gen1/2 = fresh bentonite samples, ut = untouched samples, 30D = 30-days naturally incubated sample in suspended form and K - co-isolated kit controls (also listed at the end of each sample name).


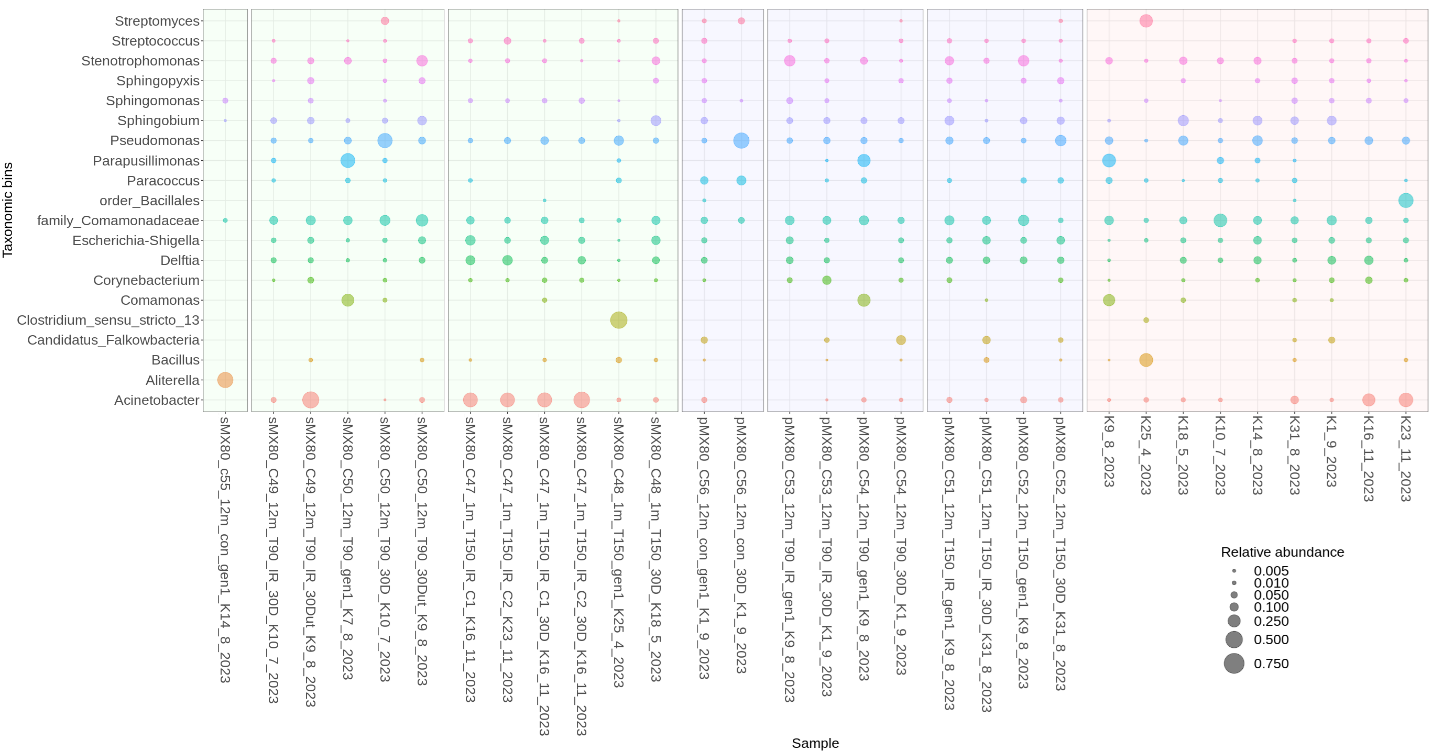


Figure S6: Microbial composition (ASV grouped at genus level) of MX-80 samples (experimental set C) from the long-term experiment. Only genera at or above 0.5% relative abundance are shown. Sample names indicate the treatment and bentonite type: sMX-80/pMX-80 - suspension/powder samples, C - sample number, 1 - 12m - exposure duration in months, T - temperature (90/150), IR - irradiated samples, con - control samples, gen1 = fresh bentonite samples, 30D = 30-days naturally incubated sample in suspended form and K - co-isolated kit controls (also listed at the end of each sample name).

Table S1: Experimental conditions used in the long-term experiment from sets A, B, and C. Sample names indicate the treatment and bentonite type (BCV/MX-80 bentonite): cBCV/MX-80 - compacted samples, pBCV/MX-80 - powder samples, sBCV/MX-80 suspension samples, C - sample number, 6-18m - exposure duration, 90/150 = applied temperature, T - temperature, IR - irradiated samples, control - control samples. The table further details the type of reactors/device and metal coupons used, saturation and resaturation conditions, dry density, and initial and final moisture content.

| Cell/ Sample number | Sample name | Set | Type of reactor/ device | Metal coupons | Saturation conditions | Dry density | Resaturation | Initial moisture content | Final moisture content | Sample type | Duration | | Temp | IR |  |
| --- | --- | --- | --- | --- | --- | --- | --- | --- | --- | --- | --- | --- | --- | --- | --- |
| 20 | cBCV_C20_6m_control | A | Stainless steel mini-module (30x30) | Carbon steel | Yes - continual | 1.6 g/cm^3^ | no | 20% | 22.58% | cBCV | 6m | | control |  |  |
| 21 | cBCV_C21_9m_control | A | Stainless steel mini-module (30x30) | Carbon steel | Yes - continual | 1.6 g/cm^3^ | no | 20% | 24.54% | cBCV | 9m | | control |  |  |
| 22 | cBCV_C22_12m_control | A | Stainless steel mini-module (30x30) | Carbon steel | Yes - continual | 1.6 g/cm^3^ | no | 20% | 23.77% | cBCV | 12m | | control |  |  |
| 23 | cBCV_C23_18m_control | A | Stainless steel mini-module (30x30) | Carbon steel | Yes - continual | 1.6 g/cm^3^ | no | 20% | 23.94% | cBCV | 18m | | control |  |  |
| 12 | cBCV_C12_9m_T90_IR | A | Stainless steel mini-module (30x30) | Carbon steel | Yes - continual | 1.6 g/cm^3^ | no | 20% | 20.86% | cBCV | 9m | | T90 | IR |  |
| 14 | cBCV_C14_12m_T90_IR | A | Stainless steel mini-module (30x30) | Carbon steel | Yes - continual | 1.6 g/cm^3^ | no | 20% | 23.24% | cBCV | 12m | | T90 | IR |  |
| 16 | cBCV_C16_18m_T90_IR | A | Stainless steel mini-module (30x30) | Carbon steel | Yes - continual | 1.6 /cm^3^ | no | 20% | 18.34% | cBCV | 18m | | T90 | IR |  |
| 13 | cBCV_C13_9m_T90 | A | Stainless steel mini-module (30x30) | Carbon steel | Yes - continual | 1.6 g/cm^3^ | no | 20% | 24.55% | cBCV | 9m | | T90 |  |  |
| 15 | cBCV_C15_12m_T90 | A | Stainless steel mini-module (30x30) | Carbon steel | Yes - continual | 1.6 g/cm^3^ | no | 20% | 23.93% | cBCV | 12m | | T90 |  |  |
| 17 | cBCV_C17_18m_T90 | A | Stainless steel mini-module (30x30) | Carbon steel | Yes - continual | 1.6 g/cm^3^ | no | 20% | 21.78% | cBCV | 18m | | T90 |  |  |
| 1 | cBCV_C1_6m_T150_IR | A | Stainless steel mini-module (30x30) | Carbon steel | Yes - continual | 1.6 g/cm^3^ | no | 15% | 21.14% | cBCV | 6m | | T150 | IR |  |
| 3 | cBCV_C3_9m_T150_IR | A | Stainless steel mini-module (30x30) | Carbon steel | Yes - continual | 1.6 g/cm^3^ | no | 15% | 25.06% | cBCV | 9m | | T150 | IR |  |
| 5 | cBCV_C5_12m_T150_IR | A | Stainless steel mini-module (30x30) | Carbon steel | Yes - continual | 1.6 g/cm^3^ | no | 15% | 20.54% | cBCV | 12m | | T150 | IR |  |
| 7 | cBCV_C7_18m_T150_IR | A | Stainless steel mini-module (30x30) | Carbon steel | Yes - continual | 1.6 g/cm^3^ | no | 15% | 22.61% | cBCV | 18m | | T150 | IR |  |
| 2 | cBCV_C2_6m_T150 | A | Stainless steel mini-module (30x30) | Carbon steel | Yes - continual | 1.6 g/cm^3^ | no | 15% | 21.28% | cBCV | 6m | | T150 |  |  |
| 4 | cBCV_C4_9m_T150 | A | Stainless steel mini-module (30x30) | Carbon steel | Yes - continual | 1.6 g/cm^3^ | no | 15% | 21.96% | cBCV | 9m | | T150 |  |  |
| 6 | cBCV_C6_12m_T150 | A | Stainless steel mini-module (30x30) | Carbon steel | Yes - continual | 1.6 g/cm^3^ | no | 15% | 9.29% | cBCV | 12m | | T150 |  |  |
| 8 | cBCV_C8_18m_T150 | A | Stainless steel mini-module (30x30) | Carbon steel | Yes - continual | 1.6 g/cm^3^ | no | 15% | 22.33% | cBCV | 18m | | T150 |  |  |
| 33 | cBCV_C33_12m_control | B | Stainless steel mini-module (30x15) | no | Partial | 1.6 g/cm^3^ | no | 15% | 11.80% | cBCV | 12m | | control |  |  |
| 32 | cBCV_C32_12m+6_control | B | Stainless steel mini-module (30x15) | no | Partial | 1.6 g/cm^3^ | yes | 15% | 24.27% | cBCV | 12+6m | | control |  |  |
| 29 | cBCV_C29_12m_T90_IR | B | Stainless steel mini-module (30x15) | no | Partial | 1.6 g/cm^3^ | no | 15% | 12.48% | cBCV | 12m | | T90 | IR |  |
| 28 | cBCV_C28_12+6m_T90_IR | B | Stainless steel mini-module (30x15) | no | Partial | 1.6 g/cm^3^ | yes | 15% | 24.34% | cBCV | 12+6m | | T90 | IR |  |
| 31 | cBCV_C31_12m_T90 | B | Stainless steel mini-module (30x15) | no | Partial | 1.6 g/cm^3^ | no | 15% | 14.05% | cBCV | 12m | | T90 |  |  |
| 30 | cBCV_C30_12m+6_T90 | B | Stainless steel mini-module (30x15) | no | Partial | 1.6 g/cm^3^ | yes | 15% | 24.19% | cBCV | 12+6m | | T90 |  |  |
| 25 | cBCV_C25_12m_T150_IR | B | Stainless steel mini-module (30x15) | no | Partial | 1.6 g/cm^3^ | no | 15% | 10.09% | cBCV | 12m | | T150 | IR |  |
| 24 | cBCV_C24_12m+6_T150_IR | B | Stainless steel mini-module (30x15) | no | partial at beginning | 1.6 g/cm^3^ | yes | 15% | 23.65% | cBCV | 12+6m | | T150 | IR |  |
| 27 | cBCV_C27_12m_T150 | B | Stainless steel mini-module (30x15) | no | Partial | 1.6 g/cm^3^ | no | 15% | 9.88% | cBCV | 12m | | T150 |  |  |
| 26 | cBCV_C26_12m+6_T150 | B | Stainless steel mini-module (30x15) | no | Partial | 1.6 g/cm^3^ | yes | 15% | 23.21% | cBCV | 12+6m | | T150 |  |  |
| 45 | sBCV_C45_12m_control | C | Glass ampoule sealed | no |  | suspension 1:3 | no | suspension 1:3 | Suspended | sBCV | 12m | | control |  |  |
| 37 | sBCV_C37_1m_T150_IR | C | Glass ampoule sealed | no |  | suspension 1:3 | no | suspension 1:3 | Suspended | sBCV | 1m | | T150 | IR |  |
| 38 | sBCV_C38_1m_T150 | C | Glass ampoule sealed | no |  | suspension 1:3 | no | suspension 1:3 | Suspended | sBCV | 1m | | T150 |  |  |
| 39 | sBCV_C39_12m_T90_IR | C | Glass ampoule sealed | no |  | suspension 1:3 | no | suspension 1:3 | Suspended | sBCV | 12m | | T90 | IR |  |
| 40 | sBCV_C40_12m_T90 | C | Glass ampoule sealed | no |  | suspension 1:3 | no | suspension 1:3 | Suspended | sBCV | 12m | | T90 |  |  |
| 46 | pBCV_C46_12m_control | C | Glass ampoule sealed | no |  | powder | no | 11.40% | Powder | pBCV | 12m | | control |  |  |
| 43 | pBCV_C43_12m_T90_IR | C | Glass ampoule sealed | no |  | powder | no | 11.40% | Powder | pBCV | 12m | | T90 | IR |  |
| 44 | pBCV_C44_12m_T90 | C | Glass ampoule sealed | no |  | powder | no | 11.40% | Powder | pBCV | 12m | | T90 |  |  |
| 41 | pBCV_C41_12m_T150_IR | C | Glass ampoule sealed | no |  | powder | no | 11.40% | Powder | pBCV | 12m | | T150 | IR |  |
| 42 | pBCV_C42_12m_T150 | C | Glass ampoule sealed | no |  | powder | no | 11.40% | Powder | pBCV | 12m | | T150 |  |  |
| 11 | cMX-80_C11_18m_control | A | Stainless steel mini-module (30x30) | Carbon steel | Yes - continual | 1.6 g/cm9 | no | 15% | 19.58% | cMX-80 | 18m | | control |  |  |
| 18 | cMX-80_C18_18m_T90_IR | A | Stainless steel mini-module (30x30) | Carbon steel | Yes - continual | 1.6 g/cm9 | no | 20% | 20.57% | cMX-80 | 18m | | T90 | IR |  |
| 19 | cMX-80_C19_18m_T90 | A | Stainless steel mini-module (30x30) | Carbon steel | Yes - continual | 1.6 g/cm9 | no | 20% | 20.42% | cMX-80 | 18m | | T90 |  |  |
| 9 | cMX-80_C9_18m_T150_IR | A | Stainless steel mini-module (30x30) | Carbon steel | Yes - continual | 1.6 g/cm9 | no | 15% | 16.57% | cMX-80 | 18m | | T150 | IR |  |
| 10 | cMX-80_C10_18m_T150 | A | Stainless steel mini-module (30x30) | Carbon steel | Yes - continual | 1.6 g/cm9 | no | 15% | 20.00% | cMX-80 | 18m | | T150 |  |  |
| 36 | cMX-80_C36_12m_control | B | Stainless steel mini-module (30x15) | no | Partial | 1.6 g/cm9 | no | 15% | 13.19% | cMX-80 | 12m | | control |  |  |
| 34 | cMX-80_C34_12m_T90_IR | B | Stainless steel mini-module (30x15) | no | Partial | 1.6 g/cm9 | no | 15% | 12.46% | cMX-80 | 12m | | T90 | IR |  |
| 35 | cMX-80_C35_12m_T90 | B | Stainless steel mini-module (30x15) | no | Partial | 1.6 g/cm9 | no | 15% | 12.54% | cMX-80 | 12m | | T90 |  |  |
| 55 | sMX-80_C55_12m_Control | C | Glass ampoule sealed | no |  | suspension 1:3 | no | suspension 1:6 | Suspended | sMX-80 | 12m | | Control |  |  |
| 49 | sMX-80_C49_12m_T90_IR | C | Glass ampoule sealed | no |  | suspension 1:3 | no | suspension 1:6 | Suspended | sMX-80 | 12m | | T90 | IR |  |
| 50 | sMX-80_C50_12m_T90 | C | Glass ampoule sealed | no |  | suspension 1:3 | no | suspension 1:6 | Suspended | sMX-80 | 12m | | T90 |  |  |
| 47 | sMX-80_C47_1m_T150_IR | C | Glass ampoule sealed | no |  | suspension 1:3 | no | suspension 1:6 | Suspended | sMX-80 | 1m | | T150 | IR |  |
| 48 | sMX-80_C48_1m_T150 | C | Glass ampoule sealed | no |  | suspension 1:3 | no | suspension 1:6 | Suspended | sMX-80 | 1m | | T150 |  |  |
| 56 | pMX-80_C56_12m_Control | C | Glass ampoule sealed | no |  | powder | no | 7.38% | Powder | pMX-80 | 12m | | Control |  |  |
| 53 | pMX-80_C53_12m_T90_IR | C | Glass ampoule sealed | no |  | powder | no | 7.38% | Powder | pMX-80 | 12m | | T90 | IR |  |
| 54 | pMX-80_C54_12m_T90 | C | Glass ampoule sealed | no |  | powder | no | 7.38% | Powder | pMX-80 | 12m | | T90 |  |  |
| 51 | pMX-80_C51_12m_T150_IR | C | Glass ampoule sealed | no |  | powder | no | 7.38% | Powder | pMX-80 | 12m | | T150 | IR |  |
| 52 | pMX-80_C52_12m_T150 | C | Glass ampoule sealed | no |  | powder | no | 7.38% | Powder | pMX-80 | | 12m | | T150 |  |

Table S3: Cq threshold values for BCV bentonite - calculation based on normalized (by sample mass) Cq values of samples from long-term (set A, B, and C) and additional experiments with all negative (by microscopy) enrichment cultures, separate calculations for bentonite samples and R2A and PGM media. Sample names indicate the treatment: cBCV/pBCV - compacted/powder samples, C - sample number, 1-18m - exposure duration in months, 90/150 = applied temperature, T - temperature, IR - irradiated samples. Gen(1/2) - fresh bentonite samples, 30D - 30-days naturally incubated sample in suspended form, AE (1/2) - R2A aerobic medium, ANA (1/2) - R2A anaerobic medium, PGM (1/2) - Postgate medium. The conservative threshold of positivity was set as Cq_Avg_ - 3xSD_Avg_ based on Westgard rules. Cq values lower than this threshold are considered positive and vice versa.

| Cell/ Sample number | Sample name |  | Bentonite | | |  | R2A media (Aerobic and Anaerobic) | | | |  | PGM1 media | |
| --- | --- | --- | --- | --- | --- | --- | --- | --- | --- | --- | --- | --- | --- |
|  |  | Set | Gen1 qPCR | Gen2 qPCR | 30D qPCR |  | AE1 qPCR | AE2 qPCR | ANA1 qPCR | ANA2 qPCR |  | PGM1 qPCR | PGM2 qPCR |
| 1 | cBCV_C1_6m_T150_IR | A | 37.84 | 32.31 | 31.20 |  | 30.10 | 34.01 | 29.66 | 29.57 |  | 33.58 | 28.41 |
| 13 | cBCV_C13_9m_T90 | A | 34.68 | 29.84 | 32.21 |  | 29.77 | 29.71 | 33.62 | 32.74 |  | 30.32 | 31.48 |
| 14 | cBCV_C14_12m_T90_IR | A | 29.39 | 29.77 | 28.33 |  | 29.18 | 29.29 | 27.32 | 28.77 |  | 26.23 | 27.57 |
| 15 | cBCV_C15_12m_T90 | A | 29.40 | 31.35 | 30.08 |  | 29.22 | 29.51 | 29.17 | 29.35 |  | 29.05 | 29.10 |
| 25 | cBCV_C25_12m_T150_IR | B | 30.90 | 30.51 | 30.75 |  | 30.63 | 30.24 | 29.43 | 29.25 |  | 28.92 | 29.13 |
| 27 | cBCV_C27_12m_T150 | B | 32.10 | 31.21 | 30.28 |  | 29.42 | 29.74 | 29.19 | 29.15 |  | 28.91 | 28.70 |
| 29 | cBCV_C29_12m_T90_IR | B | 27.27 | 29.94 | 28.33 |  | 29.53 | 28.70 | 29.07 | 29.24 |  | 28.36 | 28.34 |
| 31 | cBCV_C31_12m_T90 | B | 30.93 | 32.04 | 31.78 |  | 30.28 | 28.66 | 28.88 | 30.15 |  | 28.52 | 28.32 |
| 24 | cBCV_C24_12m+6_T150_IR | B | 30.90 | 26.85 | 30.79 |  | 28.26 | 29.28 | 29.32 | 27.73 |  | 29.20 | 29.54 |
| 26 | cBCV_C26_12m+6_T150 | B | 30.64 | 31.40 | 30.88 |  | 29.78 | 27.38 | 27.23 | 29.57 |  | 28.68 | 29.77 |
| 28 | cBCV_C28_12+6m_T90_IR | B | 30.20 | 29.72 | 30.04 |  | 28.29 | 28.30 | 20.15 | 28.90 |  | 28.66 | 27.70 |
| 30 | cBCV_C30_12m+6_T90 | B | 31.16 | 33.39 | 28.45 |  | 28.84 | 28.73 | 29.65 | 28.26 |  | 28.64 | 29.53 |
| NA | pBCV_2_1m_T90 | Additional | 30.82 | NA | 37.54 |  | 29.26 | 29.00 | 27.56 | 30.40 |  | 26.92 | 28.59 |
| NA | pBCV_1_6m_T90 | Additional | 29.41 | NA | 30.01 |  | 29.70 | 29.83 | 29.52 | 28.94 |  | 27.49 | 29.63 |
| NA | pBCV_1_1m_T150 | Additional | 31.52 | NA | 34.77 |  | 31.89 | 29.93 | 34.89 | 32.68 |  | 27.25 | 31.10 |
| NA | pBCV_2_1m_T150 | Additional | 31.64 | NA | 32.47 |  | 29.92 | 29.79 | 35.70 | 30.04 |  | 29.78 | 31.65 |
| NA | pBCV_2_3m_T150 | Additional | 29.88 | NA | 35.29 |  | 28.89 | 28.72 | 29.22 | 29.00 |  | 31.65 | 31.65 |
| NA | pBCV_1_6m_T150 | Additional | 31.01 | NA | 30.02 |  | 26.70 | 28.64 | 27.85 | 29.02 |  | 27.97 | 27.75 |
|  |  | Westgard rules | Bentonite |  |  |  | R2A |  |  |  |  | PGM |  |
|  |  | Cq_Avg_ | 31.07 |  |  |  | 29.43 |  |  |  |  | 29.11 |  |
|  |  | 1SD | 2.15 | 28.91 |  |  | 1.93 | 27.5 |  |  |  | 1.51 | 27.6 |
|  |  | 2SD | 4.31 | 26.76 |  |  | 3.86 | 25.57 |  |  |  | 3.02 | 26.09 |
|  |  | 3SD = threshold | 6.46 | 24.61 |  |  | 5.8 | 23.64 |  |  |  | 4.53 | 24.58 |

Table S4: Cq threshold values for MX-80 bentonite - calculation based on normalized (by sample mass) Cq values of samples from long-term (set A, B, and C) with all negative (by microscopy) enrichment cultures, separate calculations for bentonite samples and R2A and PGM media. Sample names indicate the treatment: cMX-80/sMX-80/pMX-80 - compacted/suspension/powder samples, C - sample number, 12-18m - exposure duration in months, 90/150 = applied temperature, T - temperature, IR - irradiated samples. Gen(1/2) - fresh bentonite samples, 30D - 30-days naturally incubated sample in suspended form, AE (1/2) - R2A aerobic medium, ANA (1/2) - R2A anaerobic medium, PGM (1/2) - Postgate medium. The conservative threshold of positivity was set as Cq_Avg_ - 3xSD_Avg_ based on Westgard rules. Cq values lower than this threshold are considered positive and vice versa.

| Cell/Sample number | Sample name |  | Bentonite | | |  | R2A media (Aerobic and Anaerobic) | | | |  | PGM media | |
| --- | --- | --- | --- | --- | --- | --- | --- | --- | --- | --- | --- | --- | --- |
|  |  | Set | Gen1 qPCR | Gen2 qPCR | 30D qPCR |  | AE1 qPCR | AE2 qPCR | ANA1 qPCR | ANA2 qPCR |  | PGM1 qPCR | PGM2 qPCR |
| 19 | cMX-80_C19_18m_T90 | A | 31.27 | NA | 31.33 |  | 28.95 | 29.10 | 29.10 | 29.29 |  | 28.18 | 27.64 |
| 9 | cMX-80_C9_18m_T150_IR | A | 30.23 | NA | 30.71 |  | 29.67 | 29.12 | 27.33 | 29.62 |  | 29.35 | 28.08 |
| 10 | cMX-80_C10_18m_T150 | A | 29.64 | NA | 31.22 |  | NA | 29.53 | 29.38 | 29.52 |  | 28.04 | 27.81 |
| 34 | cMX-80_C34_12m_T90_IR | B | 30.71 | 31.44 | 32.82 |  | 27.95 | 28.95 | 29.57 | 29.36 |  | 29.20 | 29.98 |
| 35 | cMX-80_C35_12m_T90 | B | 31.03 | 30.52 | 30.84 |  | 28.35 | 28.93 | 28.44 | 28.45 |  | 32.84 | 29.29 |
| 49 | sMX-80_C49_12m_T90_IR | C | 31.97 | NA | 34.81 |  | 28.42 | 28.79 | 28.97 | 29.38 |  | 28.62 | 29.09 |
| 50 | sMX-80_C50_12m_T90 | C | 29.63 | NA | 30.51 |  | 28.88 | 29.07 | 29.67 | 29.54 |  | 29.07 | 28.92 |
| 47 | sMX-80_C47_1m_T150_IR | C | 31.38 | NA | 31.66 |  | 29.45 | 28.24 | 29.01 | 27.99 |  | 28.10 | 27.73 |
| 48 | sMX-80_C48_1m_T150 | C | 30.26 | NA | 34.88 |  | 27.69 | 29.81 | 26.01 | 27.87 |  | 30.12 | 33.78 |
| 53 | pMX-80_C53_12m_T90_IR | C | 32.59 | NA | 30.02 |  | 29.77 | 29.42 | 30.12 | 28.41 |  | 29.84 | 28.94 |
| 54 | pMX-80_C54_12m_T90 | C | 32.71 | NA | 30.67 |  | 30.20 | 29.34 | 28.81 | NA |  | 29.91 | 29.61 |
| 51 | pMX-80_C51_12m_T150_IR | C | 32.20 | NA | 30.83 |  | 30.62 | 29.71 | 29.17 | 29.77 |  | 29.57 | 29.69 |
| 52 | pMX-80_C52_12m_T150 | C | 31.71 | NA | 31.07 |  | 34.78 | 29.54 | 29.65 | 29.06 |  | 29.11 | 28.54 |
|  |  |  |  |  |  |  |  |  |  |  |  |  |  |
|  |  | Westgard rules | Bentonite |  |  |  | R2A |  |  |  |  | PGM |  |
|  |  | Cq_Avg_ | 31.38 |  |  |  | 29.15 |  |  |  |  | 29.27 |  |
|  |  | 1SD | 1.27 | 30.12 |  |  | 1.13 | 28.03 |  |  |  | 1.38 | 27.89 |
|  |  | 2SD | 2.53 | 28.85 |  |  | 2.25 | 26.9 |  |  |  | 2.76 | 26.51 |
|  |  | 3SD = threshold | 3.8 | 27.58 |  |  | 3.38 | 25.78 |  |  |  | 4.13 | 25.13 |

Table S5: List of contaminant taxa in fresh bentonite control samples (BCV/MX-80) obtained by statistical identification using the Decontam R package.

| Bentonite control | | | | | |
| --- | --- | --- | --- | --- | --- |
| Kingdom | Phylum | Class | Order | Family | Genus |
| d_Bacteria | Proteobacteria | Gammproteobacteria | Pseudomonadales | Pseudomonadaceae | Thiopseudomonas |
| d_Bacteria | Proteobacteria | Gammproteobacteria | Pseudomonadales | Moraxellaceae | Acinetobacter |
| d_Bacteria | Proteobacteria | Gammproteobacteria | Xanthomonadales | Xanthomonadaceae | Stenotrophomonas |
| d_Bacteria | Proteobacteria | Gammproteobacteria | Enterobacterales | Enterobacteriaceae | Escherichia-Shigella |
| d_Bacteria | Actinobacteriota | Actinobacteria | Corynebacteriales | Corynebacteriaceae | Corynebacterium |
| d_Bacteria | Proteobacteria | Gammproteobacteria | Burkholderiales | Comamonadaceae | Delftia |
| d_Bacteria | Proteobacteria | Alphaproteobacteria | Rhodobacterales | Rhodobacteraceae | Paracoccus |
| d_Bacteria | Firmicutes | Bacilli | Lactobacillales | Lactobacillaceae | Lactobacillus |
| d_Bacteria | Proteobacteria | Gammproteobacteria | Pseudomonadales | Moraxellaceae | Enhydrobacter |
| d_Bacteria | Proteobacteria | Gammproteobacteria | Burkholderiales | Comamonadaceae | Comamonas |
| d_Bacteria | Proteobacteria | Gammproteobacteria | Oceanospirillales | Alcanivoracaceae1 | Alcanivorax |
| d_Bacteria | Proteobacteria | Gammproteobacteria | Pseudomonadales | Pseudomonadaceae | Pseudomonas |

Table S6: List of contaminant taxa in BCV positive enrichment cultures obtained by statistical identification using the Decontam R package.

| BCV cultivations | | | | | |
| --- | --- | --- | --- | --- | --- |
| Kingdom | Phylum | Class | Order | Family | Genus |
| d_Bacteria | Proteobacteria | Gammproteobacteria | Burkholderiales | Oxalobacteraceae | NA |
| d_Bacteria | Deinococcota | Deinococci | Thermales | Thermaceae | Thermus |
| d_Bacteria | Proteobacteria | Gammproteobacteria | Pseudomonadales | Pseudomonadaceae | Thiopseudomonas |
| d_Bacteria | Proteobacteria | Gammproteobacteria | Pseudomonadales | Pseudomonadaceae | Pseudomonas |
| d_Bacteria | Bacteroidota | Bacteroidia | Flavobacteriales | Weeksellaceae | Moheibacter |
| d_Bacteria | Proteobacteria | Gammproteobacteria | Pseudomonadales | Moraxellaceae | Acinetobacter |
| d_Bacteria | Firmicutes | Bacilli | Lactobacillales | Lactobacillaceae | Lactobacillus |
| d_Bacteria | Actinobacteriota | Actinobacteria | Corynebacteriales | Corynebacteriaceae | Corynebacterium |
| d_Bacteria | Proteobacteria | Gammproteobacteria | Burkholderiales | Neisseriaceae | NA |
| d_Bacteria | Proteobacteria | Gammproteobacteria | Enterobacterales | Enterobacteriaceae | Escherichia-Shigella |
| d_Bacteria | Proteobacteria | Gammproteobacteria | Burkholderiales | Comamonadaceae | NA |
| d_Bacteria | Proteobacteria | Gammproteobacteria | Xanthomonadales | Xanthomonadaceae | Pseudoxanthomonas |
| d_Bacteria | Actinobacteriota | Actinobacteria | Micrococcales | Micrococcaceae | Micrococcus |
| d_Bacteria | Firmicutes | Desulfotomaculia | Desulfotomaculales | Desulfotomaculales | Desulfofarcimen |
| d_Bacteria | Bacteroidota | Bacteroidia | Flavobacteriales | Flavobacteriaceae | Flavobacterium |

Table S7: List of contaminant taxa in MX-80 positive enrichment cultures obtained by statistical identification using the Decontam R package.

|  |  | MX-80 Cultivations |  |  |  |
| --- | --- | --- | --- | --- | --- |
| Kingdom | Phylum | Class | Order | Family | Genus |
| d_Bacteria | Proteobacteria | Gammproteobacteria | Pseudomonadales | Pseudomonadaceae | Pseudomonas |
| d_Bacteria | Proteobacteria | Alphaproteobacteria | Rhodobacterales | Rhodobacteraceae | Paracoccus |
| d_Bacteria | Proteobacteria | Gammproteobacteria | Burkholderiales | Comamonadaceae | NA |
| d_Bacteria | Proteobacteria | Gammproteobacteria | Enterobacterales | Enterobacteriaceae | NA |

Table S8: The Analysis of variance (ANOVA) - differences in microbial composition between treated and control bentonite samples (Control_treated), fresh/30D/corrP samples (SampleType), experimental set A, B and C (Set) and irradiated and control samples (Irradiation)

|  | BCV | | | | |  | MX-80 | | | | |  |
| --- | --- | --- | --- | --- | --- | --- | --- | --- | --- | --- | --- | --- |
|  | Df | SumOfSqs | R2 | F | Pr(>F) | Significance | Df | SumOfSqs | R2 | F | Pr(>F) | Significance |
| Control_treated | 1 | 5.122 | 0.14382 | 17.3427 | 0.001 | *** | 1 | 0.7670 | 0.06601 | 3.6032 | 0.001 | *** |
| SampleType | 2 | 1.400 | 0.03930 | 2.36.96 | 0.001 | *** | 2 | 0.8429 | 0.07254 | 1.9797 | 0.009 | ** |
| Set | 4 | 2.143 | 0.06017 | 1.8138 | 0.002 | ** | 4 | I.78 | 0.17192 | 2.3461 | 0.001 | *** |
| Irradiation | 1 | 0.368 | 0.01035 | 1.2477 | 0.199 |  | 1 | 0.3489 | 0.03002 | 1.6388 | 0.080 . |  |
| Residual | 90 | 26.579 | 0.74636 |  |  |  | 36 | 7.6636 | 0.65951 |  |  |  |
| Total | 98 | 35.611 | 1.00000 |  |  |  | 44 | 11.6201 | 1.00000 |  |  |  |

Significance codes: 0 ‘***’ 0.001 ‘**’ 0.01 ‘*’ 0.05 ‘.’ 0.1 ‘ ’ 1
